# A demethylation-activated fluorescent DNA aptamer strategy for visualising DNA alkylation repair in living cells

**DOI:** 10.1039/d6sc01299j

**Published:** 2026-05-28

**Authors:** Xinyu Luan, Han Zhang, Zhe Li, Miao Ma, Junrui Zhang, Fang Liu, Junqiu Zhai, Tiangang Luan

**Affiliations:** a State Key Laboratory of Biocontrol, School of Life Sciences, Sun Yat-sen University Guangzhou 510275 China cesltg@mail.sysu.edu.cn; b Key Laboratory of Chinese Medicinal Resource from Lingnan, Ministry of Education, School of Pharmaceutical Sciences, Guangzhou University of Chinese Medicine Guangzhou 510006 China jqzhai@gzucm.edu.cn; c School of Pharmaceutical Sciences, Sun Yat-Sen University Guangzhou 510006 China; d School of Environmental and Chemical Engineering, Wuyi University Jiangmen 529020 China; e Guangdong Provincial Laboratory of Chemistry and Fine Chemical Engineering Jieyang Center Jieyang 522000 China

## Abstract

Profiling the spatiotemporal dynamics of DNA demethylases is critical for deciphering the mechanisms of epigenetic regulation and genomic maintenance. However, existing fluorescent strategies often suffer from false-positive signals in living cells, primarily arising from non-specific nuclease degradation or instability of the complex amplification components. Herein, we present a generalisable “demethylation-activated” fluorescent light-up DNA aptamer (FLAP) strategy for high-contrast imaging of DNA alkylation repair in living cells. Our design relies on a precise “caging” strategy: site-specific methyl lesions (*e.g.*, O^6^-meG) are engineered into the ligand-binding domain of the Bibb Lettuce aptamer, which disrupts its folding and suppresses fluorescence. Upon specific enzymatic repair, the aptamer structure is restored, triggering a robust fluorescence “turn-on” signal. This mechanism effectively minimises false-positive signals. The optimised probe detects MGMT activity with high sensitivity (LOD: 1.64 nM) and enables direct visualisation of active demethylation processes in MCF-7 cells, revealing distinct responses to inhibitors. Highlighting the modularity of the platform, we extended the design to detect AlkBH2 (LOD: 0.81 nM) simply by substituting the lesion with 1-methyladenine (1-meA). This work establishes a versatile and programmable framework for converting transient DNA repair events into quantifiable optical signals, providing a powerful tool for exploring epigenetic dynamics and cancer pharmacology.

## Abstract 2

High-contrast visualisation of cellular DNA alkylation repair is realised by a demethylation-activated fluorescent DNA aptamer platform, featuring MGMT as a typical direct reversal repair target.

## Graphical abstract

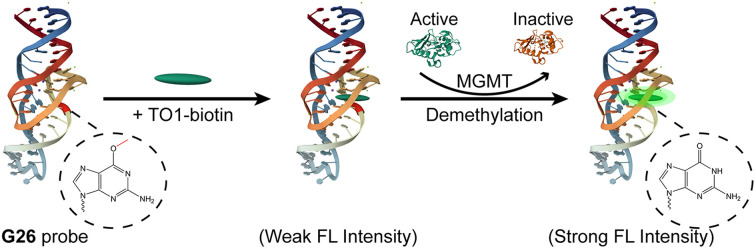

## 1 Introduction

Deciphering the mechanisms of genomic maintenance is fundamental to understanding both carcinogenesis and therapeutic resistance.^1,2^ The chemical integrity of DNA is ceaselessly challenged by alkylating agents, which introduce cytotoxic lesions such as O^6^-methylguanine (O^6^-meG) and 1-methyladenine (1-meA).^3^ The cellular response to these “chemical assaults” relies on specialised repair machinery that executes elegant, yet mechanistically distinct, reversal strategies. O^6^-Methylguanine-DNA methyltransferase (MGMT) operates *via* a unique “suicide” mechanism, directly transferring the alkyl group to a cysteine residue in a stoichiometric manner,^4^ whereas AlkBH2 utilises an oxidative demethylation pathway to restore nucleobase fidelity.^5^ Crucially, the overexpression of these repair enzymes in tumours creates a formidable barrier to chemotherapy, driving resistance to alkylating agents like temozolomide.^6^ Therefore, the ability to dynamically profile these enzymatic activities—rather than merely quantifying static protein levels—is imperative for dissecting epigenetic regulation and stratifying patients for precision cancer therapy.

Traditional methodologies, such as western blotting and ELISA, quantify protein abundance but fail to reflect the actual functional status of repair enzymes.^7,8^ To address this, fluorescent nucleic acid probes have emerged,^9–12^ employing strategies like fluorescent nucleobase analogues,^13,14^ DNAzyme activation,^15,16^ or conformational switches.^17–19^ Despite their progress, these approaches frequently suffer from false-positive signals stemming from non-specific nuclease degradation or off-target interactions.^20^ Furthermore, many existing designs rely on auxiliary enzymes (*e.g.*, exonucleases or nicking enzymes) for signal amplification,^21,22^ introducing complex variables that compromise reliability in complex biological matrices. Consequently, the development of integrated, single-molecule probes capable of high-contrast detection without reliance on auxiliary enzymes remains a critical yet unmet challenge in visualising epigenetic dynamics.

Fluorescent light-up aptamers (FLAPs), which activate small-molecule chromophores upon proper folding analogous to the mechanism of GFP, offer a promising solution to this challenge.^23–26^ Among them, the DNA aptamer Lettuce exhibits superior nuclease resistance compared to RNA-based counterparts (*e.g.*, Spinach), making it ideal for intracellular applications.^27,28^ Crucially, Lettuce's fluorescence is exquisitely sensitive to the integrity of specific nucleobases within its G-quadruplex core—a structural vulnerability that presents a unique opportunity to modulate aptamer function through site-specific chemical modifications.^29^ Bibb Lettuce, a truncated variant of the Lettuce aptamer with an identical conserved dye-binding core with Lettuce, was used in the fluorescent experiment.^29^

**Fig. 1:**
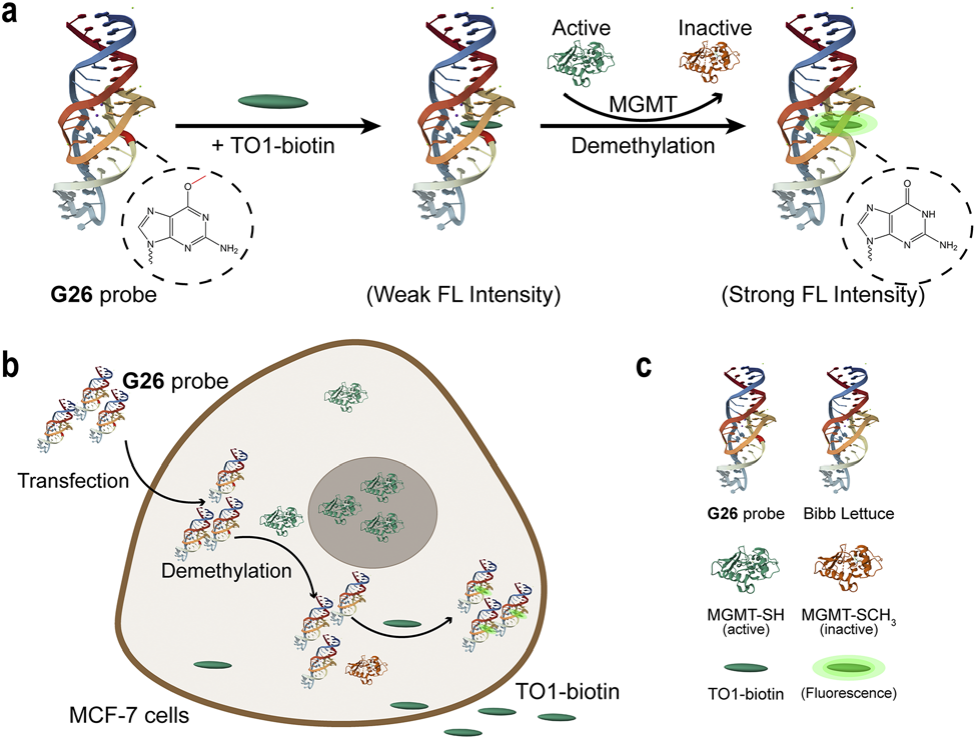
DNA alkylation repairing process detection assay through O^6^-meG-labelled FLAP. (a) Schematic diagram of G26 probe for detecting MGMT activity. (b) Schematic diagram of cell imaging experiment for MGMT activity. (c) Figure legend. The images were redrawn using PDB ID: 8FHX,^29^ 1EH6 and 1EH7.^30^

Herein, we introduce a generalisable “demethylation-activated” strategy that converts transient enzymatic repair events into robust optical signals (Fig. 1). Our design relies on a precise “caging” strategy: guided by the crystal structure of the Lettuce-fluorophore complex (PDB: 8FHX),^29^ we identified structurally critical guanine residues and engineered site-specific O^6^-meG modifications to disrupt aptamer folding and suppress fluorescence. Through systematic screening, we identified position G26 as the optimal modification site, yielding a probe with an exceptional signal-to-background ratio. Molecular dynamics simulations revealed that this high-performance stems from G26's critical role in stabilising the ligand-binding pocket through hydrogen bonding networks. The optimised probe detects MGMT activity with high sensitivity (LOD: 1.64 nM) and enables direct visualisation of active demethylation processes in living MCF-7 cells. To further validate the chemical generality of our platform, we demonstrated that the design could be readily adapted to recognise the mechanistically distinct enzyme AlkBH2 (LOD: 0.81 nM) simply by substituting the lesion with 1-methyladenine (1-meA). This work establishes a versatile and programmable framework for probing DNA repair mechanisms, offering a powerful tool for high-throughput inhibitor screening and activity-based epigenetic profiling.

## 2 Experimental

### 2.1 Reagents and materials

Oligonucleotides were synthesised by Accurate Biology (Changsha, China) and Sangon Biotech Co., Ltd (Shanghai, China). Human recombinant O^6^-methylguanine-DNA-alkyltransferase (MGMT), DFHBI-1T, O^6^-benzylguanine (O^6^-BG), tamoxifen (TAM), and bafilomycin A1 (Baf A1) were purchased from MedChemExpress (Monmouth Junction, NJ, USA). AlkB homolog 2 (AlkBH2), fat mass and obesity-associated protein (FTO) were bought from Active Motif (Carlsbad, USA). Exonuclease I (Exo I), exonuclease III (Exo III), T4 DNA ligase, and apurinic/apyrimidinic endonuclease 1 (APE1) were obtained from New England Biolabs (Ipswich, MA, USA). 1 M HEPES Buffer (pH 7.2–7.4, DNase & RNase free) was purchased from Coolaber (Beijing, China). TO1-3PEG-biotin (TO1-biotin) was obtained from ABM (Richmond, BC, Canada). KCl and MgCl_2_·6H_2_O were obtained from Guangzhou Chemical Reagent Factory (Guangzhou, China). DMSO, Fe(NH_4_)_2_(SO_4_)_2_·6H_2_O, α-ketoglutarate (α-KG), and l-ascorbic acid were obtained from Aladdin (Shanghai, China). Bovine serum albumin (BSA) was purchased from Servicebio (Wuhan, China).

DFHBI-1T was pre-prepared as a 1 M solution in DMSO prior to use, while TO1-biotin was provided in a DMF solution of 0.5 mg mL^−1^. All the other solutions used in fluorescent experiments were prepared using ultrapure water (DNase & RNase-free, sterile) obtained from Beyotime (Shanghai, China).

FuGENE was purchased from Promega Corporation (Madison, WI, USA). Opti-MEM, PBS, MitoTracker Red CMXRos, Lipofectamine 3000, and trypsin–EDTA were obtained from Thermo Fisher Scientific (Carlsbad, CA, USA). Hoechst 33342 (100×) was purchased from Biosharp (Hefei, China). Human MGMT cDNA ORF cDNA clone expression plasmid (pCMV3-untagged) and human MGMT qPCR primer pair were obtained from Sino Biological (Beijing, China). Human breast cancer cells (MCF-7, with STR analysis), minimum essential medium (MEM, with NEAA), fetal bovine serum (FBS), penicillin–streptomycin, and recombinant human insulin were gained from Procell Life Science & Technology Co., Ltd (Wuhan, China).

### 2.2 MGMT activity assay

Before the experiments, oligo DNA was added into the reaction buffer (50 mM HEPES, 140 mM KCl, 5 mM MgCl_2_, pH 7.4), heated to 95 °C for 5 min, 55 °C for 5 min, 37 °C for 5 min, then slowly cooled to room temperature (20 °C) to form the aptamer structures.

For MGMT detection, 200 nM O^6^-meG labelled Bibb Lettuce was pre-mixed with 50 µg mL^−1^ BSA and 400 nM TO1-biotin in the reaction buffer. The well-mixed solution was then incubated with MGMT of different concentrations. For the control group and blank group, no MGMT was added, and the O^6^-meG-labelled aptamer was replaced with wild type aptamer (w.t.) and no aptamer, respectively.

The fluorescence was recorded in 20 µL solution with a time interval of 1 min in a 384-well black microplate on a Tecan SPARK (Switzerland) at room temperature, with the fluorescence spectrum (*Ex* = 500 nm) recorded after 1 hour.

### 2.3 Microscopy confocal imaging

MCF-7 cells were plated on a 96-well glass-bottom plate in 0.1 mL culture medium at 37 °C for 40 h. 0.1% DMSO was added to the culture medium to dissolve additives. To investigate the effect of inhibitors on MGMT activity, 1 µM O^6^-BG or 25 µM tamoxifen were added into culture medium, respectively.

DNA transfection was performed using the commercial transfection agent FuGENE. 1 µM annealed G26 probe was mixed with 3 µL FuGENE in 20 µL Opti-MEM for 20 min at room temperature, then diluted to 100 µL, followed by adding into cell-containing wells and incubating for 3 h at 37 °C. Cells were then washed with 1× PBS to remove the untransfected aptamer. Subsequently, 0.1 mL PBS containing 10 µg mL^−1^ Hoechst 33 342 and 1 µM TO1-biotin was added into the wells and incubated for 15 min. After staining, cells were washed with 1× PBS again, and then incubated with Opti-MEM. All PBS and Opti-MEM used for transfection and staining processes were added with 5 mM MgCl_2_. For the positive control group and blank group, G26 aptamer was replaced with Bibb Lettuce aptamer (w.t.) and no aptamer, respectively.

Cell images were taken using a scanning confocal microscope (Leica TCS SP8X, Germany) at 63× magnification. The Hoechst channel was recorded under a 405 nm laser, while the TO1-biotin channel recorded under a white laser at 526 nm. Each group was imaged three times separately.

## 3 Results and discussion

### 3.1 Rational design of MGMT-responsive “caged” aptamer probes

To construct a high-performance fluorescent sensor, identifying an optimal fluorophore-aptamer pair is a prerequisite. We initiated our study by screening small-molecule chromophores, including the GFP-mimic DFHBI-1T and cyanine derivatives (TO1-biotin),^31^ against the wild-type Bibb Lettuce aptamer (Fig. S1a–c). We also tested the interactions between four compounds with similar conjugated structures (designated P1–P4; see Fig. S1d–g) and the Bibb Lettuce aptamer. The Bibb Lettuce aptamer enhanced the fluorescence of these small molecules with rotatable conjugated structures, though less effectively than with the TO1-biotin complex. While Bibb Lettuce enhanced the fluorescence of multiple candidates, TO1-biotin exhibited a superior, 65- to 70- fold fluorescence enhancement with a desirable red-shifted emission suitable for intracellular imaging (Fig. S2). Consequently, the TO1-biotin/Bibb Lettuce complex was selected as the foundational scaffold for probe engineering.

**Fig. 2:**
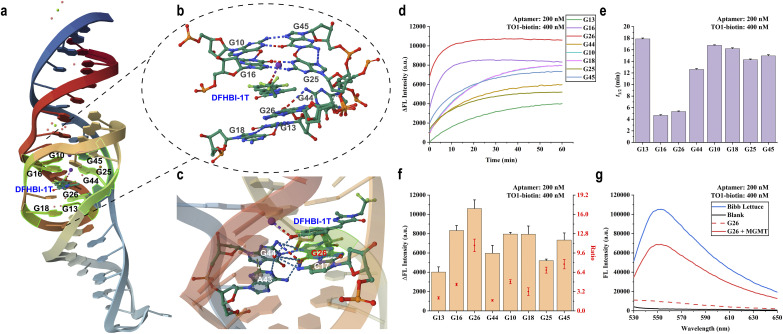
Recognition of MGMT by TO1-biotin@aptamer complexes labelled with O^6^-meG. (a) The relative positions of 8 selected guanines in the Bibb Lettuce aptamer. (b) The interactions between the 8 selected guanines and dye molecules in the Bibb Lettuce complex. (c) The interaction between the guanine nucleobase at position 26 and adjacent nucleobases, as well as dye molecules, in the Bibb Lettuce complex. (d) Fluorescence changes of O^6^-meG-labelled aptamer with MGMT (200 nM). (e) Comparison of half-life time of O^6^-meG-labelled aptamer in MGMT (200 nM) reaction. The error bars show the standard deviation (*n* = 3). (f) Fluorescence increases and proportion values of the MGMT adding group relative to the non-adding group. The error bars show the standard deviation (*n* = 3). (g) Fluorescence emission spectra of the G26 aptamer incubated for 1 hour with or without the addition of MGMT (200 nM).

Our “caging” strategy hinges on the hypothesis that site-specific O^6^-methylguanine (O^6^-meG) modifications can act as “molecular switches” to control aptamer folding. Guided by the crystal structure of the Lettuce-fluorophore complex (PDB: 8FHX),^29^ we analysed the molecular interactions governing the ligand-binding pocket. The core architecture features a G-quadruplex that anchors the chromophore through specific hydrogen bonding interactions. We identified eight strategic guanine residues located in the P1.1 and Q2 regions (G10, G13, G16, G18, G25, G26, G44, and G45) as candidate sites for modification (Fig. 2a–c). These residues are either integral to the G-quartet assembly or directly participate in ligand positioning. We posited that methylation at these positions would introduce steric hindrance or disrupt critical hydrogen bond networks, thereby enforcing an unfolded, non-fluorescent state that could be reversed solely by MGMT activity. To test this, we synthesised a library of eight O^6^-meG-modified variants (designated G10–G45) and evaluated their fluorescence response. As anticipated, the majority of the “caged” aptamers exhibited minimal fluorescence in the presence of TO1-biotin, confirming that a single methyl group is sufficient to abolish the aptamer's ability to bind and activate the fluorophore (Fig. S3b). Upon incubation with MGMT, fluorescence recovery was observed across the library, validating the enzyme's ability to access and repair lesions within structured DNA (Fig. 2d and S3a). Kinetic analysis revealed that probes modified at positions G16 and G26 displayed the most rapid repair rates, with half-lives (*t*_1/2_) of 4.65 and 5.32 min, respectively (Fig. 2e). Among these, the G26 probe emerged as the premier candidate, exhibiting the highest signal-to-background ratio and a robust ∼10-fold fluorescence enhancement (Fig. 2f). The fluorescence emission spectra (Fig. 2g and S3c–d) further support this demethylation-triggered fluorescence light-up trend.

### 3.2 Molecular mechanism of the G26 probe

To elucidate the molecular basis underlying the exceptional performance of the G26 probe, we performed MD simulations comparing the wild-type and O^6^-meG-modified Lettuce-fluorophore complexes. The crystal structure (PDB: 8FHX)^29^ served as the starting template. We constructed an *in silico* oxygen of guanine at position 26 was replaced with a methoxy group. Both systems were subjected to 80 ns production MD simulations using AMBER20 with the OL15 force field for DNA and GAFF for the fluorophore (see SI for computational details).

**Fig. 3:**
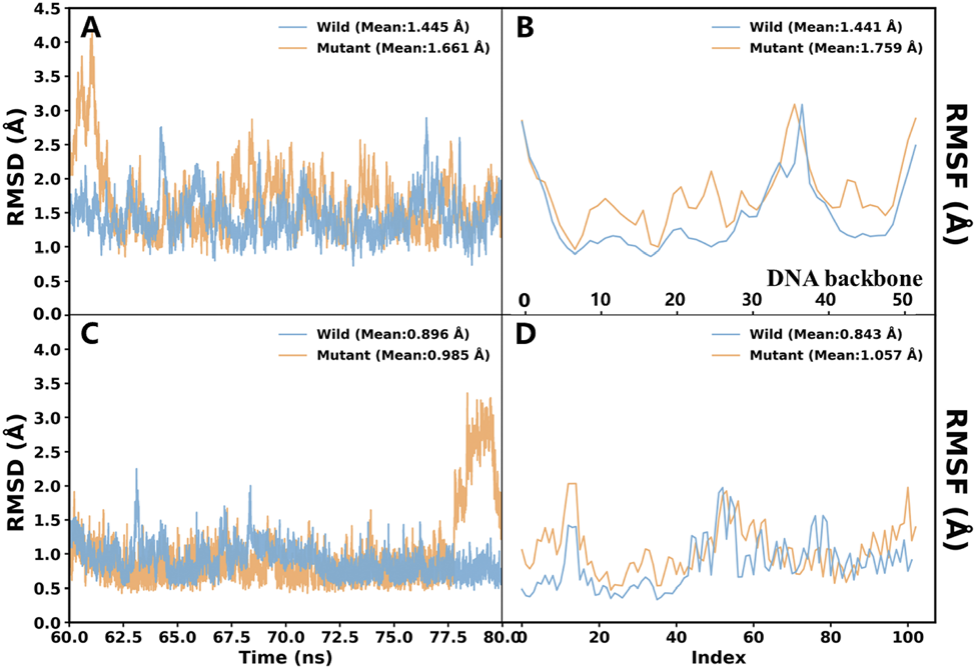
RMSD and RMSF analyses for the wild-type and mutant systems. After alignment of the trajectories to the DNA backbone atoms, RMSD time courses were calculated for (a) the DNA backbone and (c) ligand heavy atoms. The RMSF distributions for the wild-type and mutant systems include (b) RMSF of DNA backbone atoms along the sequence and (d) RMSF of ligand heavy atoms.

Analysis of the simulation trajectories revealed differences in structural dynamics between the two systems (Fig. 3). The wild-type complex maintained a stable conformation with low backbone RMSD, while the O^6^-meG-modified system (mutant) exhibited elevated RMSD values, particularly for the fluorophore relative to the DNA. RMSF analysis corroborated these findings: the fluorophore displayed significantly enhanced conformational freedom in the mutant system.

Structural interpretation of these dynamics revealed that G26 participates in critical hydrogen bonding interactions that stabilise the ligand-binding pocket. Introduction of the O^6^-methyl group sterically clashes with these partners, preventing formation of the compact tertiary structure required for stable fluorophore binding. Upon MGMT-mediated demethylation, the native guanine is restored, allowing the aptamer to spontaneously refold and activate the fluorophore through restricted non-radiative energy dissipation. Notably, these structure-switching principles extend to other fluorophores such as DFHBI-1T, underscoring the generality of our caging mechanism.

These computational findings were further confirmed by experimental circular dichroism (CD) spectroscopy and thermal melting analysis. As shown in Fig. S4, O^6^-meG modification at G26 reduced the characteristic G-quadruplex signal at 290 nm and decreased the structural stability of the G26 aptamer, while MGMT-mediated demethylation effectively restored both the G-quadruplex feature and thermal stability, corroborating the structural mechanism proposed by MD simulations.

### 3.3 *In vitro* sensitivity and specificity of the G26 probe

**Fig. 4:**
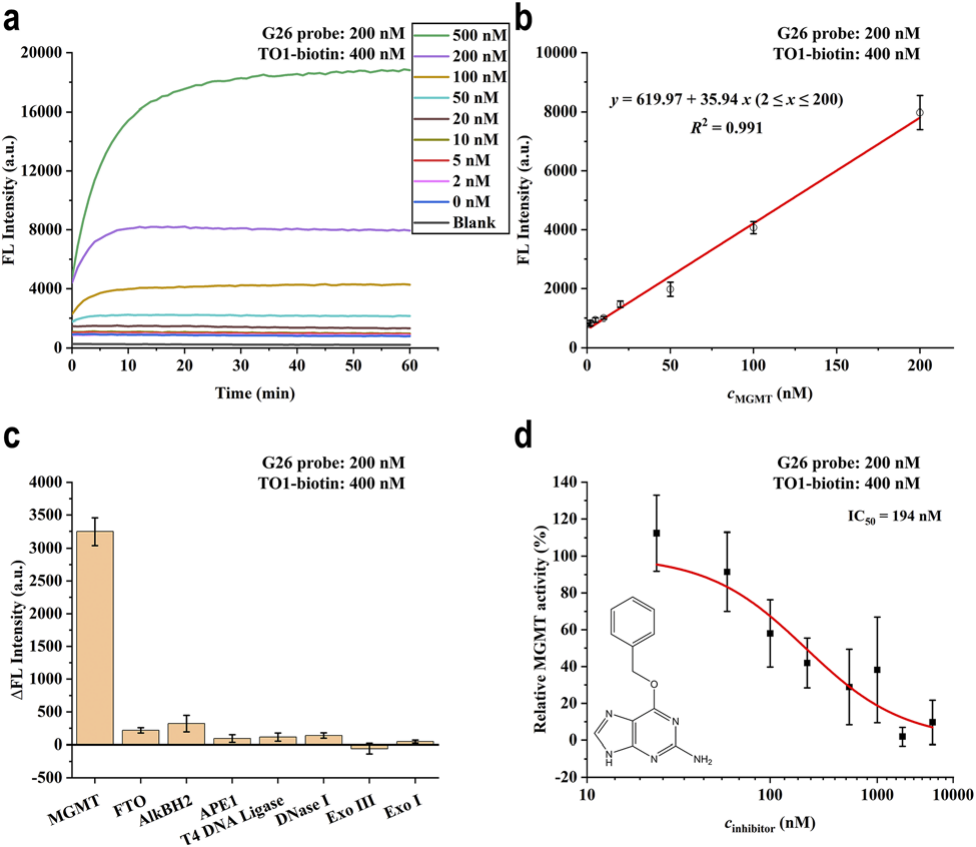
Assessment of the G26 probe for MGMT detection. (a) Fluorescence values of G26 probe at different concentrations of MGMT over the course of 1 hour. (b) The standard curve of the G26 probe with different concentrations of MGMT (2–200 nM). (c) Fluorescence signals of G26 probe to different DNA repair enzymes, compared to the group without protein addition. (MGMT/AlkBH2/FTO: 100 nM; APE1/T4 DNA ligase/DNase I/Exo III/Exo I: 10 U mL^−1^) (d) G26 evaluates the activity of inhibitor O^6^-BG. The error bars show the standard deviation (*n* = 3).

Having identified G26 as the optimal probe, we systematically characterised its analytical performance for MGMT detection. The probe exhibited a robust dose-dependent fluorescence response to MGMT (Fig. 4a and S5), with excellent linearity (*R*^2^ > 0.99) across a concentration range of 2–200 nM and a calculated limit of detection (LOD) of 1.64 nM based on the 3*σ*/slope criterion (Fig. 4b). To assess specificity, we incubated the G26 probe with a panel of DNA repair enzymes including AlkBH2, T4 DNA Ligase, FTO, APE1, and several nucleases. Remarkably, significant fluorescence enhancement was observed exclusively in the presence of MGMT, with negligible responses to other proteins (Fig. 4c). This exceptional selectivity can be attributed to the unique direct repair mechanism of MGMT, which specifically recognises and removes O^6^-alkyl lesions through an irreversible alkyl transfer reaction.

To demonstrate the utility of the probe for drug discovery applications, we evaluated its performance in screening MGMT inhibitors using O^6^-benzylguanine (O^6^-BG) as a model compound.^32^ The G26 probe successfully captured the dose-dependent inhibition of MGMT activity, yielding an IC_50_ value of 194 nM (Fig. 4d), which closely matches literature values.^33^ This validation confirms that our probe can serve as a sensitive and specific platform for high-throughput screening of MGMT inhibitors, offering significant advantages over traditional assays.

### 3.4 Live-cell imaging of endogenous MGMT activity

**Fig. 5:**
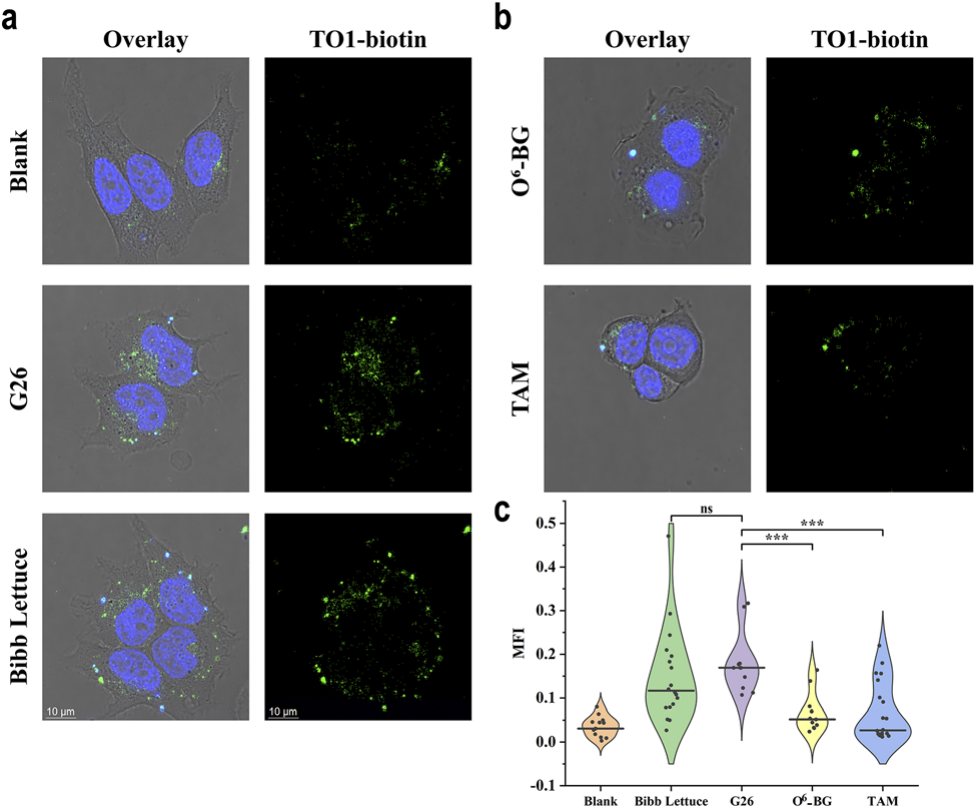
Live-cell imaging of endogenous MGMT activity using the G26 probe. (a) Representative CLSM images of MCF-7 cells. Blank: negative control group, without aptamer transfection; G26: experimental probe group, transfected with 1.0 µM G26 probe; Bibb Lettuce: positive control group, transfected with 1.0 µM wild-type Bibb Lettuce aptamer. After transfection, all groups were stained with 1 µM TO1-biotin and 10 µg mL^−1^ Hoechst 33 342 before imaging. (b) CLSM results of inhibitor treatment groups. O^6^-BG: pre-incubated with culture medium containing 1 µM O^6^-BG for 40 h; TAM: pre-incubated with culture medium containing 25 µM tamoxifen for 40 h. Both groups were transfected with 1 µM G26 probe and stained as above before imaging. (c) Quantitative analysis of single-cell fluorescence intensity across treatment groups. Data represented as median (black line) with individual data points. Statistical significance was determined by Mann–Whitney *U* test: ***, *p* < 0.001; ns, not significant (*p* > 0.05).

Encouraged by the robust of G26 probe *in vitro* performance, we investigated its capability to visualise endogenous MGMT activity within the complex intracellular environment. The human breast cancer cell line MCF-7 was selected as a model system due to its established high expression of MGMT,^34,35^ which was validated through experiments (Fig. S6). Confocal laser scanning microscopy (CLSM) was employed to monitor the fluorescence response at the single-cell level. To validate the probe's fluorogenic competency and cellular uptake, we compared the G26 probe against specific controls (Fig. 5a). Cells transfected with the wild-type Bibb Lettuce aptamer (Lettuce group) displayed bright green fluorescence, confirming efficient intracellular delivery and aptamer folding. In contrast, non-transfected cells (blank) remained dark. Crucially, cells transfected with the G26 probe exhibited distinct green fluorescence, providing direct evidence that the endogenous MGMT machinery successfully repaired the O^6^-meG lesion, thereby restoring the aptamer's structure and activating TO1-biotin fluorescence *in situ*.

To exclude potential interference from the non-specific mitochondrial association of TO1-biotin,^36,37^ we performed a co-localisation analysis using MitoTracker (Fig. S7). Following background subtraction, the values of Pearson's correlation coefficient and Manders' coefficient M2 were both below 0.1 (Table S2), indicating that the specific light-up signal shows no significant co-localisation with mitochondria. Further control experiments using bafilomycin A1 (Baf A1) demonstrated that inhibiting endosomal acidification blocked G26 from entering the cytosol, thus abolishing fluorescence activation (Fig. S8). MGMT overexpression and siRNA-mediated knockdown further confirmed that the signal response was strictly dependent on MGMT activity (Fig. S9). Changes in MGMT expression were validated by qPCR and western blot analysis (Fig. S9c–e).

To further verify that the observed signal specifically arises from MGMT activity, we performed inhibition assays using two mechanistically distinct small molecules: O^6^-benzylguanine (O^6^-BG), a pseudosubstrate that irreversibly inactivates MGMT,^32^ and tamoxifen (TAM), which promotes MGMT proteasomal degradation.^38^ As anticipated, MCF-7 cells pre-treated with either O^6^-BG or TAM showed a marked reduction in intracellular fluorescence compared to the untreated G26 group (Fig. 5b). Quantitative single-cell analysis revealed a statistically significant attenuation in mean fluorescence intensity (Fig. 5c), corroborating that the G26 probe dynamically reports on the functional status of the enzyme. Additionally, control experiments conducted with Bibb Lettuce aptamers in the presence of MGMT inhibitors did not show significant fluorescence reduction, further confirming that the fluorescence reduction was specifically dependent on MGMT demethylation activity (Fig. S10). These results demonstrate that the probe is biologically stable and sensitive enough to differentiate varying levels of enzymatic activity, positioning it as a potent tool for monitoring epigenetic repair dynamics in live cells.

### 3.5 Generalising the platform: programmable detection of AlkBH2

**Fig. 6:**
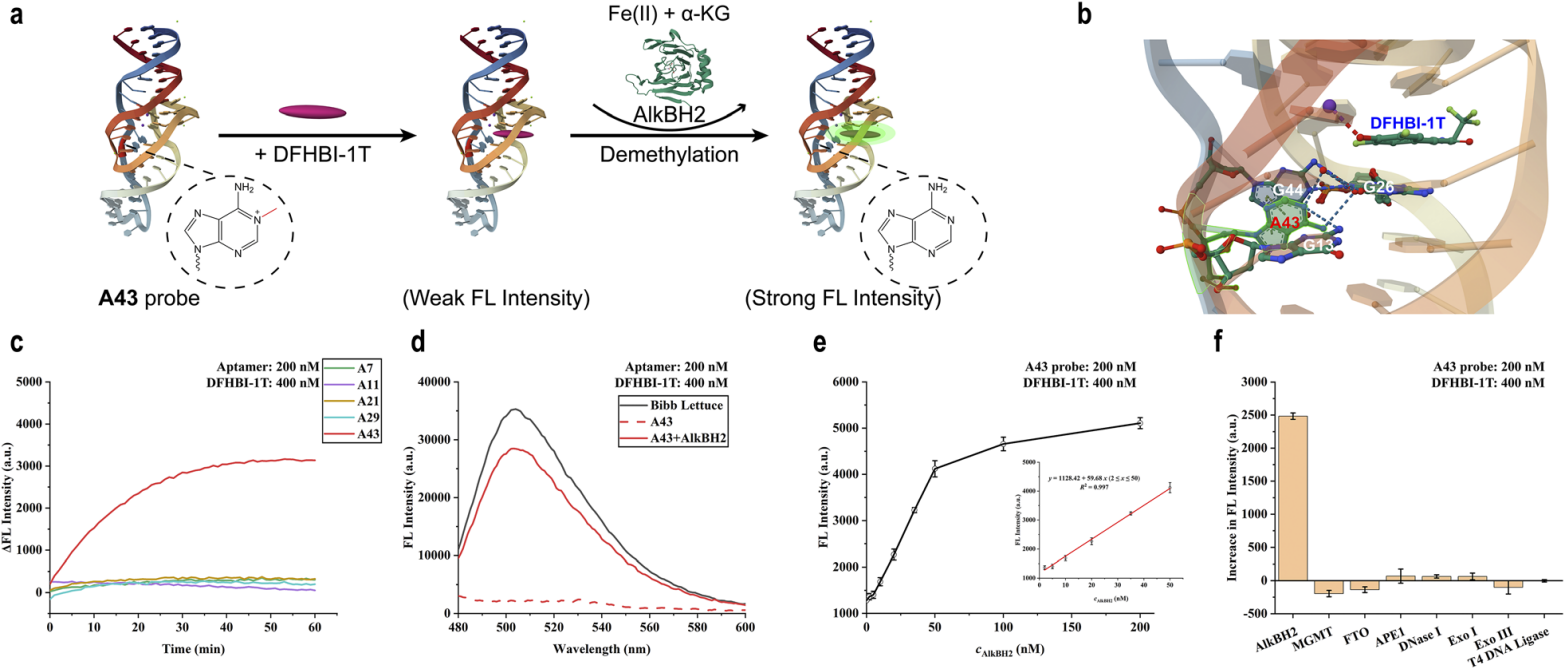
Recognition of AlkBH2 by DFHBI-1T@aptamer complexes labelled with 1-meA. (a) Schematic diagram of A43 probe for detecting AlkBH2 activity. (b) The interaction between the adenine nucleobase at position 43 and adjacent nucleobases, as well as dye molecules, in the Bibb Lettuce complex. (c) The fluorescence difference of 1-meA-labelled aptamer with AlkBH2 (100 nM) adding compared to the group without AlkBH2 within 1 hour. (d) Fluorescence emission spectra of the A43 aptamer incubated for 1 hour with or without the addition of AlkBH2 (100 nM). The spectrum has been smoothed. (e) The final fluorescence values of the A43 probe after 1 hour of interaction with AlkBH2 of different concentrations. The standard curve of the A43 probe with different concentrations of AlkBH2 (2–50 nM) was displayed on the right side of the image. (f) Fluorescence increment of A43 probe treated with different DNA repair enzymes for 1 hour. (AlkBH2/MGMT/FTO: 100 nM; APE1/T4 DNA ligase/DNase I/Exo III/Exo I: 10 U mL^−1^) the error bars show the standard deviation (*n* = 3). The images were redrawn using PDB ID: 8FHX and 3BU0.

To demonstrate the modularity and generalisability of the “demethylation-activated” strategy, we sought to reprogram the sensor to detect AlkBH2, a mechanistically distinct enzyme that repairs N1-methyladenine (1-meA) lesions (Fig. 6a). Our design principle posits that the probe's specificity can be redirected by simply substituting the chemical identity of the “caging” lesion from O^6^-meG to 1-meA while retaining the aptamer scaffold. Given the low adenine abundance in the Bibb Lettuce sequence, we systematically introduced 1-meA modifications at five available positions (A7, A11, A21, A29, and A43) to generate a library of A*x* variants (Fig. 6b and S11a). Initial screening revealed that unlike the MGMT probes, 1-meA-modified aptamers exhibited higher background fluorescence with TO1-biotin. To address this, we leveraged the fluorophore flexibility of Bibb Lettuce and switched to DFHBI-1T (Fig. 6d and S12). This strategic substitution effectively suppressed the background signal, underscoring the platform's adaptability to different reporter modules.

Subsequent screening revealed that the A43 variant uniquely functioned as a responsive sensor, displaying a robust fluorescence turn-on specifically upon AlkBH2 treatment (Fig. 6c, S11b and S13a–b). While other positions remained inert or constitutively fluorescent, the 1-meA43 aptamer (designated A43) confirmed a 5˜-fold enhancement at 503 nm (Fig. 6d and S13c–d). The unique sensitivity of position 43 suggests that this adenine residue plays an indispensable role in stabilising the aptamer's tertiary fold, likely through critical stacking interactions that are severely perturbed by the N1-methyl group (Fig. 6b).

To further evaluate the performance of the A43 probe in detecting AlkBH2, we conducted sensitivity and selectivity experiments. The results demonstrated that the A43 probe exhibits a concentration-dependent fluorescent response toward AlkBH2 (Fig. S11c and S14). This fluorescence signal exhibits a linear relationship with AlkBH2 concentration within a certain range (2–50 nM, *R*^2^ > 0.99), demonstrating a detection limit of 0.81 nM calculated by 3*σ*/slope (Fig. 6e). Subsequent experiments demonstrated that the fluorescence signal of the A43 probe exhibits high selectivity for AlkBH2 compared to other DNA repair enzymes (Fig. 6f). This successful expansion to a second enzyme target confirms that our “caging” strategy is not limited to a specific lesion or repair pathway but serves as a versatile, programmable framework for identifying diverse DNA repair activities.

Compared with previously reported fluorescent nucleic acid-based strategies for MGMT/AlkBH2 detection relying on caging DNAzyme catalytic core^33,35^ or inhibiting restriction endonuclease cleavage,^21,22^ our demethylation-activated FLAP platform directly reports DNA repair signals without the rate-limiting catalytic steps (Table S3). Although certain *in vitro* assays achieve lower MGMT detection limits with additional protein (*e.g.* DNA polymerase)^15^ or specialised metal (*e.g.* Zn^2+^)^34^ components, these approaches complicate experimental design and restrict cellular imaging applicability. Our system enables rapid detection within one hour and successful intracellular imaging for MGMT. Regarding AlkBH2 detection, we established a linear quantitative response, complementing existing qualitative or semi-quantitative tools.^13,33^

## 4 Conclusions

Overall, we developed a versatile “demethylation-activated” FLAP platform for sensitive detection of DNA alkylation repair enzymes in living cells. By engineering site-specific methyl lesions into the Bibb Lettuce aptamer, we achieved sub-nanomolar detection limits for both MGMT (1.64 nM) and AlkBH2 (0.81 nM) with exceptional specificity. By simply substituting methylation lesion types, the modular design enables detection of mechanistically distinct repair pathways through a common fluorogenic framework. Beyond analytical performance, the platform enables real-time visualisation of repair dynamics in cancer cells, screening of clinical inhibitors, and monitoring cellular responses to environmental DNA-damaging agents. These capabilities position our approach as a valuable tool bridging fundamental DNA repair research and clinical applications. This work establishes a generalisable strategy for converting DNA repair events into quantifiable fluorescent signals, validating that masking aptamer folding *via* atomic-level modifications represents a versatile methodology to regulate DNA function. We envision that this technology will not only facilitate fundamental studies of epigenetic maintenance but also accelerate the development of activity-based diagnostics for precision cancer therapy.

## Author contributions

Xinyu Luan carried out most parts of the experiments and wrote the original manuscript. Han Zhang assisted in cell culture and cellular experiments, analysed the data and provided comments. Zhe Li was responsible for the computational simulation. Miao Ma and Junrui Zhang revised the manuscript. Fang Liu provided candidate dyes and revised the manuscript. Junqiu Zhai and Tiangang Luan supervised the project, analysed the data and revised the manuscript. All authors have approved the final version of the manuscript.

## Conflicts of interest

There are no conflicts to declare.

## Supplementary Material

SC-OLF-D6SC01299J-s001

## Data Availability

All data supporting the findings of this study are available within the article. Ref. 39–46 are cited in the supplementary information (SI). Supplementary information: experimental details, computational details, oligonucleotide sequences, Tables S1–S3 and supplementary figures. Structural coordinates from RCSB PDB: 8FHX (https://doi.org/10.2210/pdb8fhx/pdb), 1EH6 (https://doi.org/10.2210/pdb1eh6/pdb), 1EH7 (https://doi.org/10.2210/pdb1eh7/pdb), 3BU0 (https://doi.org/10.2210/pdb3bu0/pdb); relevant structure graphics were generated using Mol*. See DOI: https://doi.org/10.1039/d6sc01299j.
