# Supplementary material for: A demethylation-activated fluorescent DNA aptamer strategy for visualising DNA alkylation repair in living cells
Source: Chem Sci. 2026 May 28;17(27):13598–605. doi: 10.1039/d6sc01299j (PMC13232633; doi:10.1039/d6sc01299j)
Supplement: SC-017-D6SC01299J-s001 [file SC-017-D6SC01299J-s001.pdf]

Supplementary Information (SI) for Chemical Science.
This journal is © The Royal Society of Chemistry 2026

Supporting Information

A demethylation-activated fluorescent DNA aptamer strategy for visualising DNA alkylation repair in living cells

Xinyu Luan,[a] Han Zhang,[b] Zhe Li,[c] Miao Ma,[a] Junrui Zhang,[a] Fang Liu,[b] Junqiu Zhai,*[b,a] Tiangang Luan*[a,d,e]

[a] State Key Laboratory of Biocontrol, School of Life Sciences, Sun Yat-sen University, Guangzhou 510275, China

[b] Key Laboratory of Chinese Medicinal Resource from Lingnan, Ministry of Education, School of Pharmaceutical Sciences, Guangzhou University of Chinese Medicine, Guangzhou, 510006, China

[c] State Key Laboratory of Anti-Infective Drug Discovery and Development, School of Pharmaceutical Sciences, Sun Yat-sen University, Guangzhou 510006, China

[d] School of Environmental and Chemical Engineering, Wuyi University, Jiangmen 529020, China

[e] Guangdong Provincial Laboratory of Chemistry and Fine Chemical Engineering Jieyang Center, Jieyang 522000, China

Table of contents

Experimental section...3
1. MGMT activity detection...3
2. AlkBH2 activity detection...4
3. Circular dichroism (CD) spectroscopy measurements...5
4. Cell culture...5
5. Confocal microscopy imaging...5
6. Western blot (WB) experiments...6
7. Quantitative real-time PCR (qPCR) experiments...7

Computational details...9
Table S1 The sequences of the DNA aptamers and siRNA used in the article....11
Table S2 Pearson's correlation coefficients and Manders' coefficients of co-localisation analysis12
Table S3 Comparison of analytical performance for reported MGMT/AlkBH2 detection methods. ...13
Fig. S1 The fluorescence enhancement of Bibb Lettuce aptamer on different dye molecules. ...14
Fig. S2 Effects of BSA addition and TO1-biotin concentration on the fluorescence...15
Fig. S3 Fluorescence values and fluorescence emission spectra of O^6-meG-labelled aptamer with and without the addition of MGMT within 1 hour....16
Fig. S4 The circular dichroism (CD) characterisation of the aptamers....17
Fig. S5 Fluorescence emission spectra of the **G26** probe with the addition of different concentrations of MGMT after 1 hour. ...18
Fig. S6 Expression analysis of MGMT protein in MCF-7 and BEAS-2B cells. ...19
Fig. S7 Co-localisation analysis of TO1-biotin with mitochondria. ...20
Fig. S8 Endosomal escape inhibition experiment using the **G26** probe. ...21
Fig. S9 MGMT overexpress and knockdown validation using the **G26** probe...22
Fig. S10 Inhibitor specificity control in living cells using the Bibb Lettuce aptamer....24
Fig. S11 Recognition of AlkBH2 by DFHBI-1T@aptamer complexes labelled with 1-meA. ...25
Fig. S12 Fluorescence emission spectra of the **A43** aptamer incubated with TO1-biotin for 1 hour with or without the addition of AlkBH2 (100 nM). ...26
Fig. S13 Fluorescence values and fluorescence emission spectra of 1-meA-labelled aptamer with and without the addition of AlkBH2 within 1 hour....27
Fig. S14 Fluorescence emission spectra of the **A43** probe with the addition of different concentrations of AlkBH2 after 1 hour. ...28
Reference...29

Experimental section

1. MGMT activity detection

1.1 O^6-meG position screening

200 nM Bibb Lettuce with single O^6-meG lesion at different positions (G13, G16, G26, G44, G10, G18, G25, and G45), respectively, were pre-mixed with 50 μg/mL BSA and 400 nM TO1-biotin in the reaction buffer (50 mM HEPES, 140 mM KCl, 5 mM $MgCl_2$, pH 7.4). The well-mixed solution was then incubated with 0 nM or 200 nM MGMT for reaction. For the control group and blank group, no MGMT was added, and the O^6-meG-labelled aptamer was replaced with wild type (w.t.) aptamer and no aptamer, respectively.

1.2 Sensitive assay

200 nM Bibb Lettuce with single O^6-meG lesion at G26 position (named **G26** probe) was pre-mixed with 50 μg/mL BSA and 400 nM TO1-biotin in the reaction buffer (50 mM HEPES, 140 mM KCl, 5 mM $MgCl_2$, pH 7.4). The well-mixed solution was then incubated with MGMT of different concentrations (0, 2, 5, 10, 20, 50, 100, 200, 500 nM) for reaction. For the blank group, no aptamer nor MGMT was added.

1.3 Selective assay

200 nM **G26** probe was pre-mixed with 50 μg/mL BSA and 400 nM TO1-biotin in the reaction buffer (50 mM HEPES, 140 mM KCl, 5 mM $MgCl_2$, pH 7.4). The well-mixed solution was then incubated with 10 U/mL protein (APE1, T4 DNA Ligase, DNase I, Exo III, Exo I) or 100 nM DNA demethylase (MGMT, AlkBH2), mixed with the corresponding reaction buffer. For the control group, no additional protein nor buffer was added.

1.4 Inhibitor validation

100 nM MGMT and O^6-BG of different concentrations (20, 50, 100, 200, 500, 1000, 2000, 10000 nM) were pre-mixed at room temperature in the reaction buffer for 30 min, while 200 nM **G26** probe was pre-mixed with 50 μg/mL BSA and 400 nM TO1-biotin in the reaction buffer (50 mM HEPES, 140 mM KCl, 5 mM $MgCl_2$, pH 7.4). The probe-containing mixture

was then incubated with O^6-BG-treated MGMT for reaction. For the control group, 100 nM MGMT was incubated without O^6-BG at room temperature before adding into the probe-containing mixture.

2. AlkBH2 activity detection

The annealing program was consistent with the MGMT detection method. The fluorescence was recorded in 50 μL solution with a time interval of 1 min in a 384-well black microplate on a Tecan SPARK (Switzerland) at room temperature, with fluorescence spectrum (*Ex* = 450 nm) recorded after 1 hour.

2.1 1-meA position screening

200 nM Bibb Lettuce with single 1-meA lesion at different positions (A7, A11, A21, A29, A43), respectively, were pre-mixed with 400 nM DFHBI-1T. The solution was then incubated with 0 or 100 nM AlkBH2 in reaction buffer (50 mM HEPES, 140 mM KCl, 5 mM $MgCl_2$, 50 μg/mL BSA, 75 μM $Fe(NH_4)_2(SO_4)_2$, 1 mM α-KG, 2 mM L-ascorbic acid, pH 7.4) for reaction. For the control group and blank group, no AlkBH2 was added, and 1-meA-labelled aptamer was replaced with w.t. aptamer and no aptamer, respectively.

2.2 Sensitive assay

200 nM Bibb Lettuce with single 1-meA lesion at A43 position (named **A43** probe) was pre-mixed with 400 nM DFHBI-1T. The solution was then incubated with AlkBH2 of different concentrations (0, 2, 5, 10, 20, 35, 50, 100, 200 nM) in reaction buffer (50 mM HEPES, 140 mM KCl, 5 mM $MgCl_2$, 50 μg/mL BSA, 75 μM $Fe(NH_4)_2(SO_4)_2$, 1 mM α-KG, 2 mM L-ascorbic acid, pH 7.4). For the blank group, no aptamer nor MGMT was added.

2.3 Selective assay

200 nM **A43** probe was pre-mixed with 400 nM DFHBI-1T in the basic reaction buffer (50 mM HEPES, 140 mM KCl, 5 mM $MgCl_2$, pH 7.4). The well-mixed solution was then incubated with 10 U/mL protein (APE1, T4 DNA Ligase, DNase I, Exo III, Exo I) or 100 nM DNA demethylase (MGMT, AlkBH2), mixed with the corresponding reaction buffer. For the

control group, no additional protein nor buffer was added.

3. Circular dichroism (CD) spectroscopy measurements

The CD spectra and thermal melting curves were recorded with a Chirascan plus CD spectrometer (Applied Photophysics, UK) with a 0.5 mm path-length cell. All samples (Bibb Lettuce, **G26**, and **G26** + MGMT) were prepared in the same buffer (50 mM HEPES, 140 mM KCl, 5 mM $MgCl_2$, pH 7.4) at a final concentration of 5 μM after annealing. The **G26** + MGMT group was pre-incubated with 10 μM MGMT for over 1 h prior to measurement.

CD spectra were collected from 210 to 320 nm at 1 nm intervals at 25 °C, with each spectrum averaged over five scans. For thermal melting analysis, the CD intensity was monitored as the sample was heated from 25 °C to 90 °C at a rate of 1 °C/min. Melting temperature (T_m) values were determined by non-linear Boltzmann fitting of the melting curves, using five representative data points per degree Celsius. All raw data were smoothed and baseline-corrected for subsequent analysis.

4. Cell culture

MCF-7 cells were cultured in MEM (with NEAA), supplemented with 10% FBS, 10 mg/mL insulin, and 100 U/mL penicillin-streptomycin. The cells were cultured in a humidified incubator at 37 °C with 5% CO_2.

BEAS-2B cells were cultured in DMEM (MeilunBio, Dalian, China), supplemented with 10% FBS, and 100 U/mL penicillin-streptomycin. The cells were cultured in a humidified incubator at 37 °C with 5% CO_2.

5. Confocal microscopy imaging

The basic transfection steps, staining methods, and confocal imaging settings are described in the manuscript. After 40 h of incubation, 1 μM aptamer probes were transfected into the experimental group for 3 h, while the blank group received no aptamer transfection. After staining and washing, the cells were imaged in the 405 nm (Hoechst 33342) and 526 nm (TO1-biotin) channels by confocal microscopy at 63× magnification.

For co-localisation analysis of TO1-biotin with mitochondria, 0.1 mL PBS containing 10 μg/mL Hoechst 33342, 100 nM MitoTracker Red CMXRos and 1 μM TO1-biotin was used for staining, while the MitoTracker channel was recorded under a white laser at 578 nm, independent of the TO1-biotin channel.

For endosomal escape inhibitor experiments, the **G26** probe was chosen for both control and experimental groups. Cells of the bafilomycin A1 (Baf A1) experimental group were pre-incubated with 100 nM Baf A1 for 1 h before transfection, then transfected with 1.0 μM G26 probe under Baf A1 treatment conditions.

For inhibitor specificity control experiments, the Bibb Lettuce aptamer was chosen for control and experimental groups. 1 μM O^6-BG or 25 μM tamoxifen was individually added into 0.1% DMSO-containing culture medium for 40 h before transfection with the Bibb Lettuce aptamer in experimental groups.

For regulation of MGMT expression in live cells, plasmids of human MGMT (pMGMT) were constructed in a mammalian expression vector pCMV3 to upregulate MGMT expression (HG12077-UT, Sino Biological, Beijing, China), while human MGMT-targeting siRNAs were synthesized to downregulate MGMT expression (Sangon Biotech, Shanghai, China). For confocal microscopy imaging, MCF-7 cells were seeded on 96-well glass-bottom plates in 0.1 mL of culture medium and incubated at 37 °C for 40 h. pMGMT (2 μg/mL), MGMT siRNA (50 nM) or NC (negative control) siRNA (50 nM) were mixed with FuGENE (at a ratio of 1:3) at room temperature for 15 min, then the mixture was added to 0.1 mL Opti-MEM, and this solution was subsequently added to the cells. After 6 h of incubation, the medium was replaced with fresh MCF-7 complete medium, and the cells were incubated for 18 h. Following the above pre-treatment, the cells were transfected with 1 μM annealed G26 probe at 37 °C for 3 h using the method described in the manuscript. The methods for staining and confocal imaging are the same as those described elsewhere in the manuscript.

6. Western blot (WB) experiments

To compare MGMT expression in different cell lines, MCF-7 and BEAS-2B cells were cultured in 6-well plates. For MGMT overexpression and knockdown assays, MCF7 cells were

seeded in 6-well plates with 1.5 mL of complete medium and incubated at 37 °C for 40 h. Cells were then transfected with MGMT plasmid (2 μg/mL), MGMT siRNA (50 nM) or negative control siRNA (50 nM) using FuGENE reagent (1:3) and incubated for 6 h. The culture medium was then replaced with fresh complete medium, and cells were cultured for an additional 18 h before being collected for Western blot analysis.

The cells were harvested, washed with 1× PBS and lysed using RIPA lysis buffer (Beyotime, Shanghai, China) containing protease inhibitor cocktails (Servicebio, Wuhan, China). Total protein concentration was then quantified using a BCA protein assay kit (Beyotime, Shanghai, China).

Proteins of equal concentration were separated using sodium dodecyl sulphate-polyacrylamide gel electrophoresis (SDS-PAGE) and transferred onto polyvinylidene difluoride (PVDF) membranes (Millipore, Billerica, MA, USA). The PVDF membranes were blocked with 5% non-fat milk for 1 h, then incubated overnight with diluted anti-MGMT primary antibody (MedChemExpress, Monmouth Junction, NJ, USA) at 4 °C. After being washed three times with TBST, the membranes were incubated with horseradish peroxidase-conjugated goat anti-rabbit IgG (Boster, Wuhan, China) for 1 h at room temperature. GAPDH was measured as an internal reference protein using anti-GAPDH (Affinity Bioscience, Cincinnati, OH, USA).

Signals were detected using a super ECL ultra kit (ABP Biosciences, Rockville, MD, USA).

7. Quantitative real-time PCR (qPCR) experiments

To compare MGMT expression in different cell lines, MCF-7 and BEAS-2B cells were seeded in 6-well plates in 1.5 mL of culture medium and incubated at 37 °C for about 2.5 days. For regulation of MGMT expression in living cells, MCF-7 cells were seeded in 6-well plates in 1.5 mL of culture medium and incubated at 37 °C for 40 h. pMGMT (2 μg/mL), MGMT siRNA (50 nM) or NC (negative control) siRNA (50 nM) were mixed with FuGENE or Lipofectamine 3000 (1:3) at room temperature for 15 min, then the mixture was added to 1 mL Opti-MEM, and this solution was subsequently added to the cells. After 6 h of incubation, the

medium was replaced with fresh MCF-7 complete medium, and the cells were incubated for an additional 18 h before qPCR experiments.

Then, total RNA was isolated from these cells utilising the miRNeasy Mini Kit (purchased from Qiagen, Hilden, Germany) following the manufacturer's protocol. Reverse transcription was conducted with 5× All-In-One RT MasterMix from Abm (Richmond, BC, Canada).

PCR was performed with Archimed X4 qPCR (RocGene, Beijing). The MGMT primers were purchased from Sino Biological (Cat No.101605, Beijing, China). The expression of GAPDH was measured as an internal reference gene. The GAPDH primers synthesised by Sangon (Shanghai, China) were as follows: GAPDH-qpcr-FP: 5'-GTCTCCTCTGACTTCAACAGCG-3'; GAPDH-qpcr-RP: 5'-ACCACCCTGTTGCTGTAGCCAA-3'

All experiments were performed in three independent replicates. The $2^{-\Delta\Delta Ct}$ method was used to analyse experimental data.

Computational details

In this study, the crystal structure of the DNA-ligand complex was obtained from the Protein Data Bank (PDB ID: 8FHX). The co-crystallized small-molecule ligand and crystallographic water molecules were removed, and only the DNA and key monovalent and divalent metal ions (K^{+}, Mg^{2+}) were retained. The carbonyl oxygen of guanine at position 26 was manually replaced by a methoxy group in Schrödinger Maestro, yielding an O-methyl-guanine residue. Because the fluorescent small molecule used in this study is large and highly elongated, conventional automatic docking protocols could not generate physically reasonable DNA–ligand conformations. Therefore, based on the above 8FHX-derived DNA models, the fluorescent probe was manually placed into the DNA binding site using Discovery Studio. By translating and rotating the ligand, its chromophore was adjusted to approximately occupy the binding region corresponding to the original co-crystallized ligand in 8FHX.

After obtaining the manually constructed DNA-ligand complexes, each complex was embedded in a rectangular box of TIP3P water molecules,[39] and Na^{+} or Cl^{-} ions were added to neutralize the system. Ligand restrained electrostatic potential (RESP) charges were derived at the HF/6-31G* level using Gaussian 03, and the ligand was modeled with the GAFF force field. [40] The was parameterized using the Amber OL15 force field. [41]

All molecular dynamics simulations were carried out with AMBER20. First, each system was subjected to a four-stage energy minimization. In each stage, 2500 steps of steepest descent were followed by 2500 steps of conjugate gradient minimization, while the positional restraints were gradually relaxed. Subsequently, Langevin dynamics[42,43] was employed to slowly heat each system from 0 to 300 K over 50 ps. The systems were then equilibrated in the NPT ensemble: an initial density equilibration was performed under weak restraints on the complex, followed by 100 ps of constant-pressure equilibration using the Parrinello–Rahman pressure-coupling algorithm,[44] with the pressure maintained at approximately 1 atm. During the above NVT and NPT pre-equilibration stages, the positional restraint force constant was set to 10 $kcal \cdot mol^{-1} \cdot Å^{-2}$. Thereafter, production MD simulations of 80 ns were performed for each DNA-ligand system in the NPT ensemble at 1 atm and 300 K under periodic boundary

conditions. All bonds involving hydrogen atoms were restrained using the SHAKE algorithm,[45] allowing a time step of 2 fs. Long-range electrostatic interactions were treated with the particle mesh Ewald (PME) method,[46] with a real-space cutoff of 8 Å.

To evaluate the stability of the DNA-ligand complexes, the last 20 ns of each trajectory were used for analysis. The trajectories were first aligned to the DNA backbone atoms, and the RMSD and RMSF of the DNA backbone and ligand heavy atoms were then calculated separately. As shown in **Fig. 3a** and **3c**, after alignment to the DNA backbone, the RMSD of the DNA backbone in the mutant system initially increased and then plateaued. In contrast, the wild-type system exhibited overall lower RMSD values that remained stable, suggesting a somewhat higher global stability. Meanwhile, the ligand RMSD shown in **Fig. 3c** remains consistently stable throughout the simulation in the wild-type system. In the mutant system, however, the ligand RMSD showed increased fluctuations during the final few nanoseconds of the simulation.

Fig. 3b and **3d** show the RMSF profiles of the DNA backbone and ligand heavy atoms along the sequence. Overall, most regions of the DNA duplex in both systems display low RMSF values, indicating that the global framework is relatively rigid. Notably, in the wild-type system, the local segment containing the 26th nucleotide and its neighboring bases exhibits smaller backbone fluctuations, whereas in the mutant system the RMSF of this region is elevated, suggesting that the mutation increases local flexibility and weakens structural restraints in this part of the DNA. The ligand RMSF analysis further shows that, in the mutant system, several segments of the ligand exhibit larger fluctuations than in the wild-type, indicating that the mutation increases the conformational freedom and local flexibility of the ligand within the binding site, consistent with the reduced stability of the binding mode revealed by the RMSD analysis.

Table S1 The sequences of the DNA aptamers and siRNA used in the article.

**Name**	**Sequences (5'-3')**
**Lettuce**	CTTAGTAGGGATGATGCGGCAGTGGGCTTCGCAGTTCCTGCGAG GGGACTAAG
	**Bibb Lettuce and variants**
**Bibb Lettuce (wild-type)**	CGCGAGGGGTAGGGATGTGGCGGCAGTGGGCCACGCG
G13 probe	CGCGAGGGGTAGGGAT/O^6-meG/TGGCGGCAGTGGGCCACGCG
G16 probe	CGCGAGGGGTAGGGATGTG/O^6-meG/CGGCAGTGGGCCACGCG
**G26 probe**	CGCGAGGGGTAGGGATGTGGCGGCAGTGG/O^6-meG/CCACGCG
G44 probe	CGCGA/O^6-meG/GGGTAGGGATGTGGCGGCAGTGGGCCACGCG
G10 probe	CGCGAGGGGTAGG/O^6-meG/ATGTGGCGGCAGTGGGCCACGCG
G18 probe	CGCGAGGGGTAGGGATGTGGC/O^6-meG/GCAGTGGGCCACGCG
G25 probe	CGCGAGGGGTAGGGATGTGGCGGCAGTG/O^6-meG/GCCACGCG
G45 probe	CGCGAG/O^6-meG/GGTAGGGATGTGGCGGCAGTGGGCCACGCG
A7 probe	CGCGAGGGGT/1-meA/GGGATGTGGCGGCAGTGGGCCACGCG
A11 probe	CGCGAGGGGTAGGG/1-meA/TGTGGCGGCAGTGGGCCACGCG
A21 probe	CGCGAGGGGTAGGGATGTGGCGGC/1-meA/GTGGGCCACGCG
A29 probe	CGCGAGGGGTAGGGATGTGGCGGCAGTGGGCC/1-meA/CGCG
**A43 probe**	CGCG/1-meA/GGGGTAGGGATGTGGCGGCAGTGGGCCACGCG
	**siRNA**
MGMT-sense	GAGCAGGGUCUGCACGAAAUA/dT//dT/
MGMT-antisense	UAUUUCGUGCAGACCCUGCUC/dT//dT/
NC-sense	UUCUCCGAACGUGUCACGU/dT//dT/
NC-antisense	ACGUGACACGUUCGGAGAA/dT//dT/

Table S2 Pearson's correlation coefficients and Manders' coefficients of co-localisation analysis

	Manders' M1*	Manders' M2**	Pearson's correlation coefficient (*r*)***
Blank	0.977 ± 0.014	0.029 ± 0.002	0.0653 ± 0.0112
**G26**	0.884 ± 0.025	0.062 ± 0.013	0.0502 ± 0.0064
Bibb Lettuce	0.837 ± 0.101	0.079 ± 0.013	0.0466 ± 0.0117

*M1: Fraction of TO1-biotin-Bibb Lettuce signal overlapping with MitoTracker;

**M2: Fraction of MitoTracker signal overlapping with the TO1-biotin-Bibb Lettuce;

****r*: Linear correlation coefficient between the two fluorescence signals (−1 to 1, closer to 1 = higher co-localisation).

All data represent mean ± SD from three independent fields of view after background subtraction.

Table S3 Comparison of analytical performance for reported MGMT/AlkBH2 detection methods.

	Mechanism	LOD	Linear range	Reaction Time	Cellular Applicability	Ref.
MGMT	*Pvu*II*	0.5 ng/mL	1 – 25 ng/mL	~3 h	—	[21]
MGMT	*Pvu*II	1.34 fM	1.0×10^{-8} – 0.1 ng/mL	60 min	—	[22]
MGMT	8-17 Dz**	2 nM	9 – 540 nM	3 h	Yes	[33]
MGMT	8-17 Dz	8.17×10^{-9} ng/μL	1.0×10^{-8} – 1.0×10^{-2} ng/μL	1.5 h	—	[15]
MGMT	I-R3 Dz	0.054 nM	10 – 1000 nM	Overnight	—	[34]
MGMT	10-23 Dz	4 nM	10 – 100 nM	3 h	Yes	[35]
MGMT	FLAP***	1.64 nM	2 – 200 nM	1 h	Yes	This work
AlkBH2	PCT****	—	—	Over 24 h	—	[13]
AlkBH2	8-17 Dz	—	—	2 h	Yes	[33]
AlkBH2	FLAP	0.81 nM	2 – 50 nM	1 h	—	This work

**Pvu*II: restriction enzyme (*Pvu*II) recognition site blocking;

**Dz: DNAzyme caging;

***FLAP: fluorescent light-up DNA aptamer caging;

****PCT: photo-induced charge transfer.

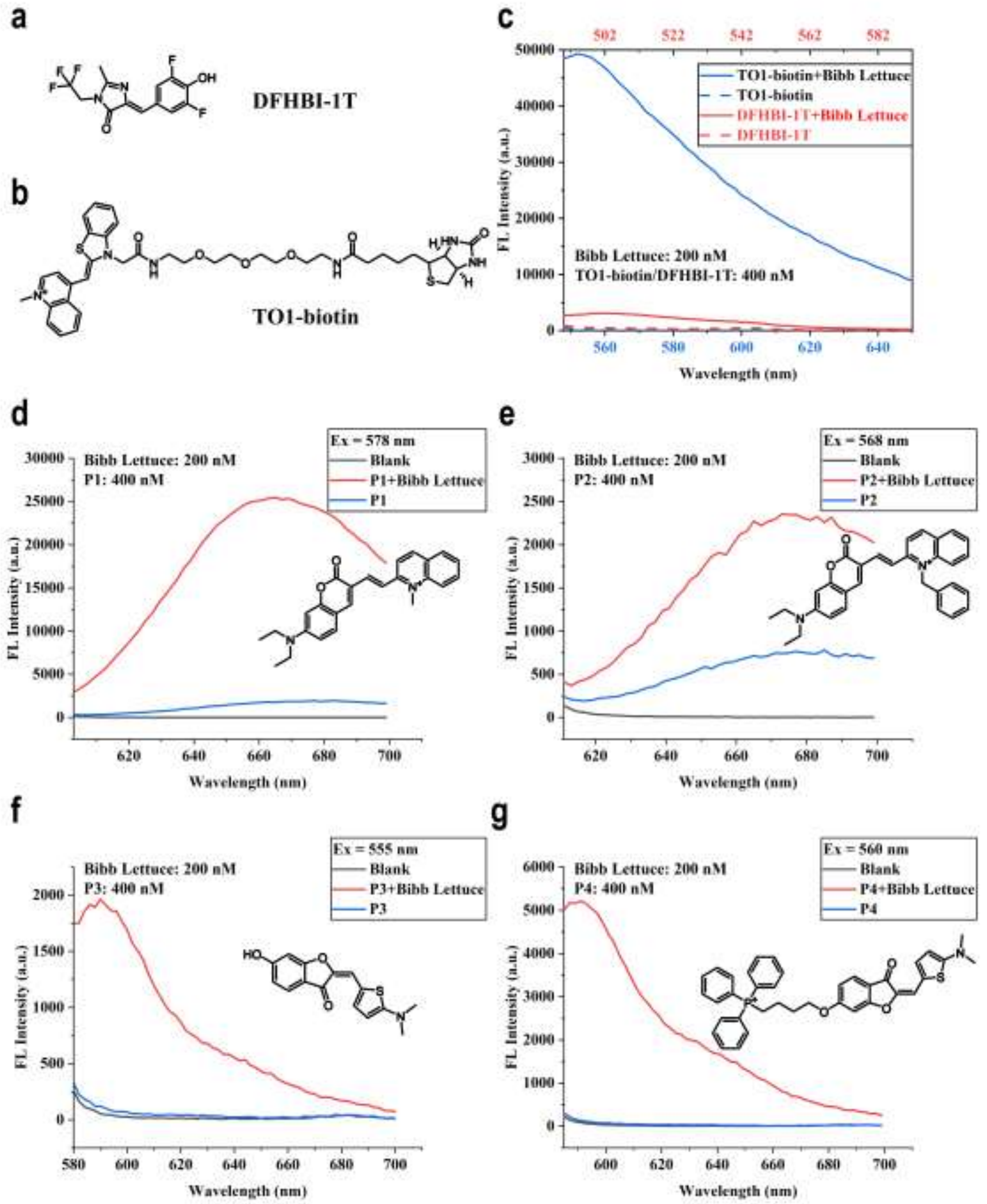

Fig. S1 The fluorescence enhancement of Bibb Lettuce aptamer on different dye molecules.

(a) The structure of DFHBI-1T ($C_{13}H_9F_5N_2O_2$); (b) The structure of TO1-biotin (TO1-3PEG-biotin, $C_{38}H_{49}N_6O_6S_2$); (c) The fluorescence enhancement of Bibb Lettuce aptamer on DFHBI-1T and TO1-biotin; (d–g) The fluorescence enhancement of P1 ($C_{25}H_{25}N_2O_2$, d), P2 ($C_{31}H_{29}N_2O_2$, e), P3 ($C_{15}H_{13}NO_3S$, f), and P4 ($C_{37}H_{35}NO_3PS$, g). The structure of P1, P2, P3, and P4 were shown in corresponding figure.

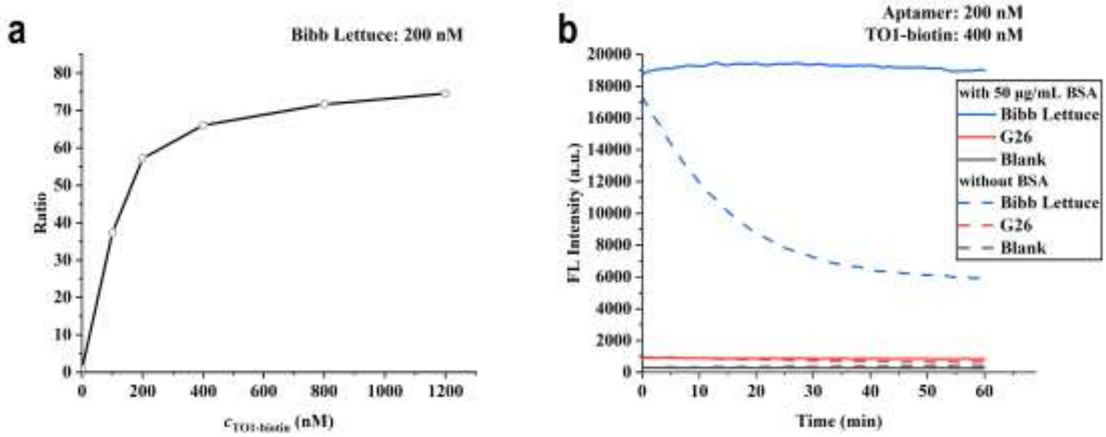

Fig. S2 Effects of BSA addition and TO1-biotin concentration on the fluorescence. (a) FL intensity ratio of TO1-biotin-Bibb Lettuce complex/TO1-biotin. (b) Comparison of the fluorescence behaviours of the TO1-biotin-Bibb Lettuce complex and the **G26** probe in the presence or absence of BSA.

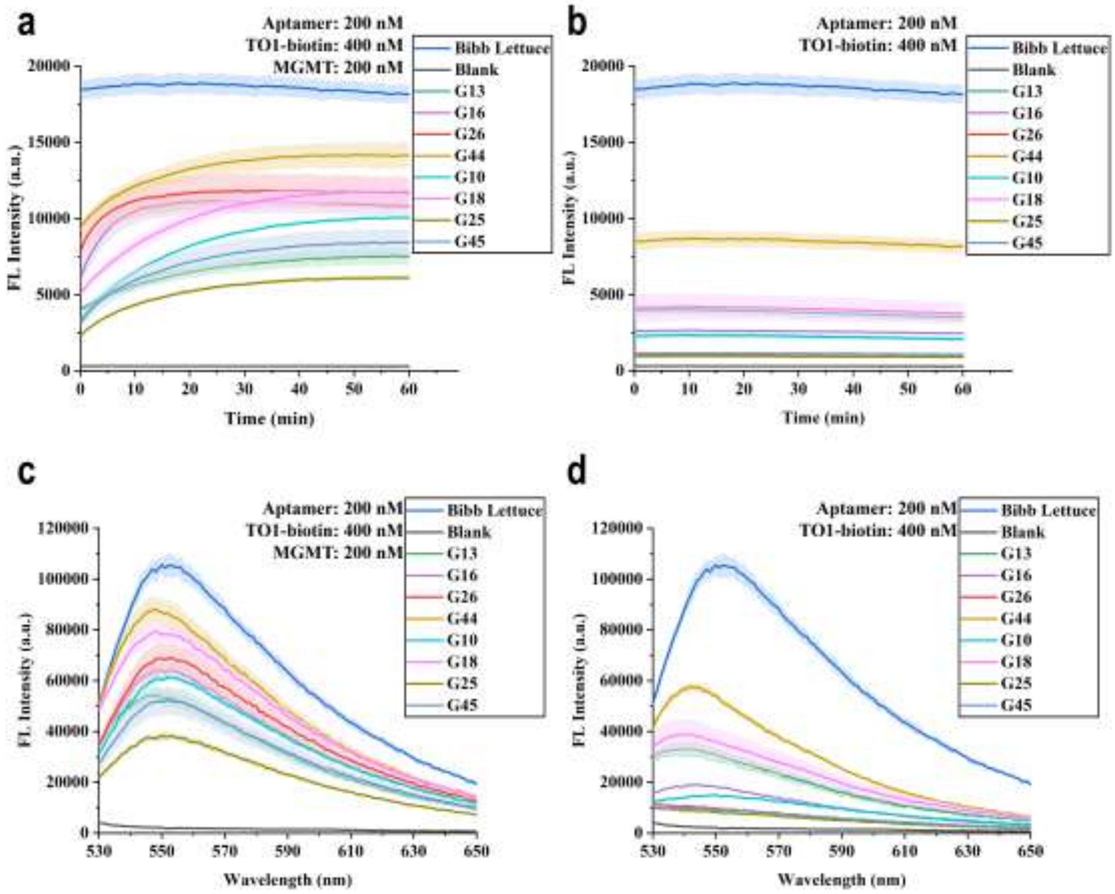

Fig. S3 Fluorescence values and fluorescence emission spectra of O^6-meG-labelled aptamer with and without the addition of MGMT within 1 hour.

(a) Fluorescence values of O^6-meG-labelled aptamer with the addition of 200 nM MGMT within 1 hour; (b) Fluorescence values of O^6-meG-labelled aptamer without the addition of MGMT within 1 hour; (c) Fluorescence emission spectra of O^6-meG-labelled aptamer with the addition of 200 nM MGMT after 1 hour; (d) Fluorescence emission spectra of O^6-meG-labelled aptamer without the addition of MGMT after 1 hour. The error bars show the standard deviation (n = 3).

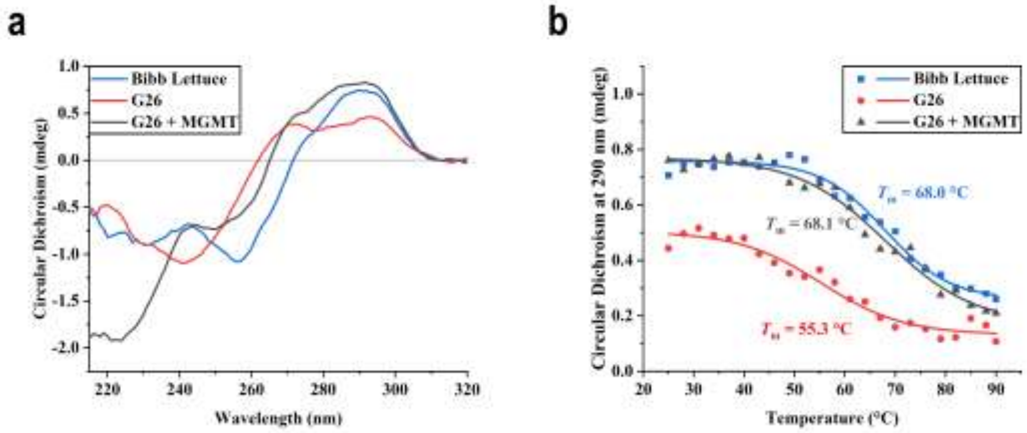

Fig. S4 The circular dichroism (CD) characterisation of the aptamers.

(a) CD spectra of Bibb Lettuce, **G26** probe, and MGMT-treated **G26** probe recorded at 25 °C; (b) CD thermal melting curves (25 – 90 °C) of Bibb Lettuce, **G26** probe, and MGMT-treated **G26** probe monitored at 290 nm with corresponding melting temperatures (T_m) labelled.

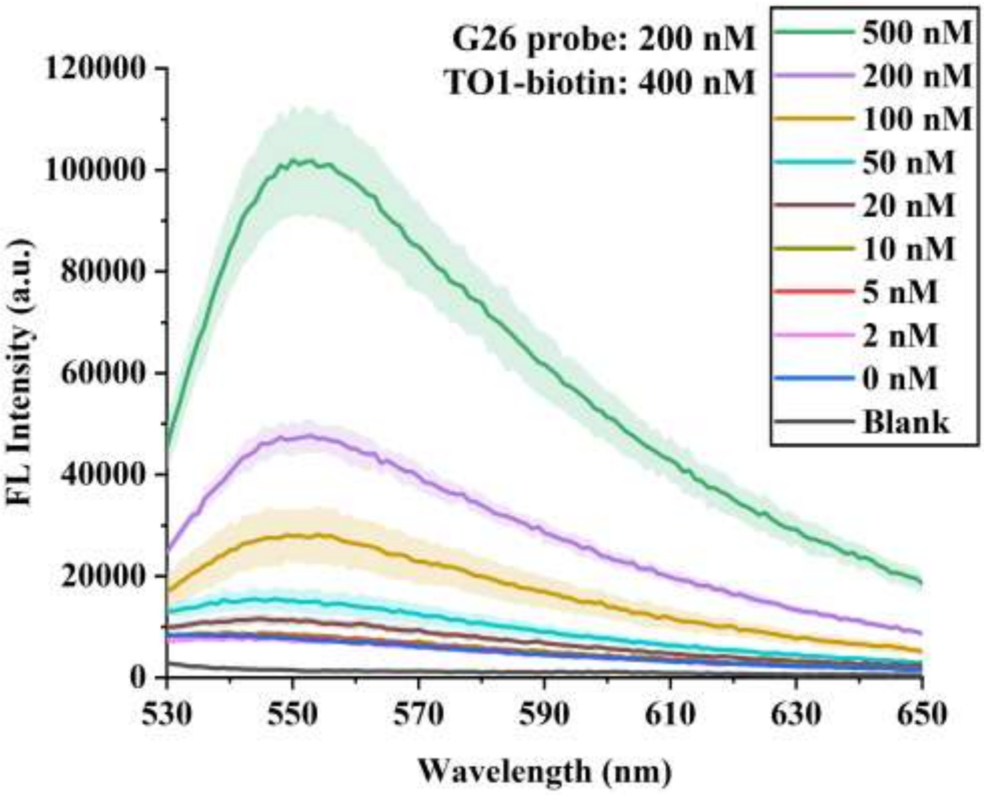

Fig. S5 Fluorescence emission spectra of the **G26** probe with the addition of different concentrations of MGMT after 1 hour.

The error bars show the standard deviation (n = 3).

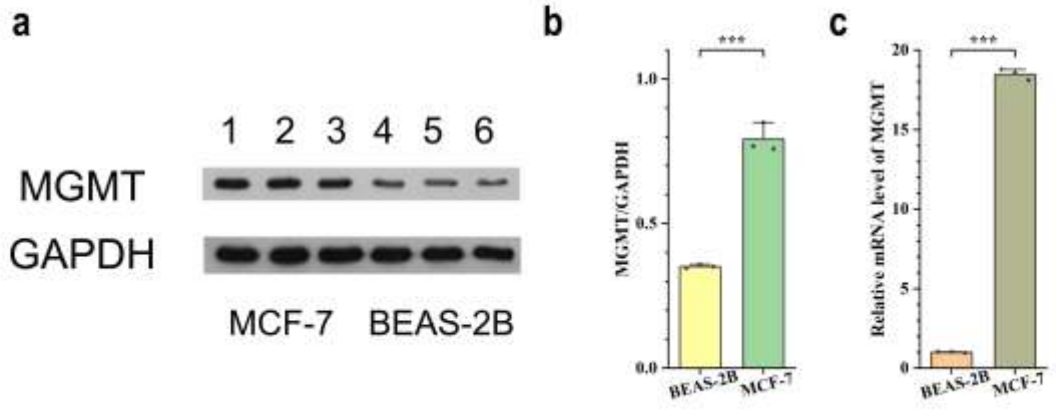

Fig. S6 Expression analysis of MGMT protein in MCF-7 and BEAS-2B cells.

(a) Representative Western blot analysis of MGMT in MCF-7 cells, with BEAS-2B normal cells as the normal control group. (b) Quantitative analysis of band intensities following densitometric quantification, normalised to GAPDH. (c) Relative MGMT mRNA levels between MCF-7 and BEAS-2B cells, normalised to GAPDH. The error bars show the standard deviation (n = 3). Statistical significance was determined by one-way ANOVA test: ***, $p < 0.001$.

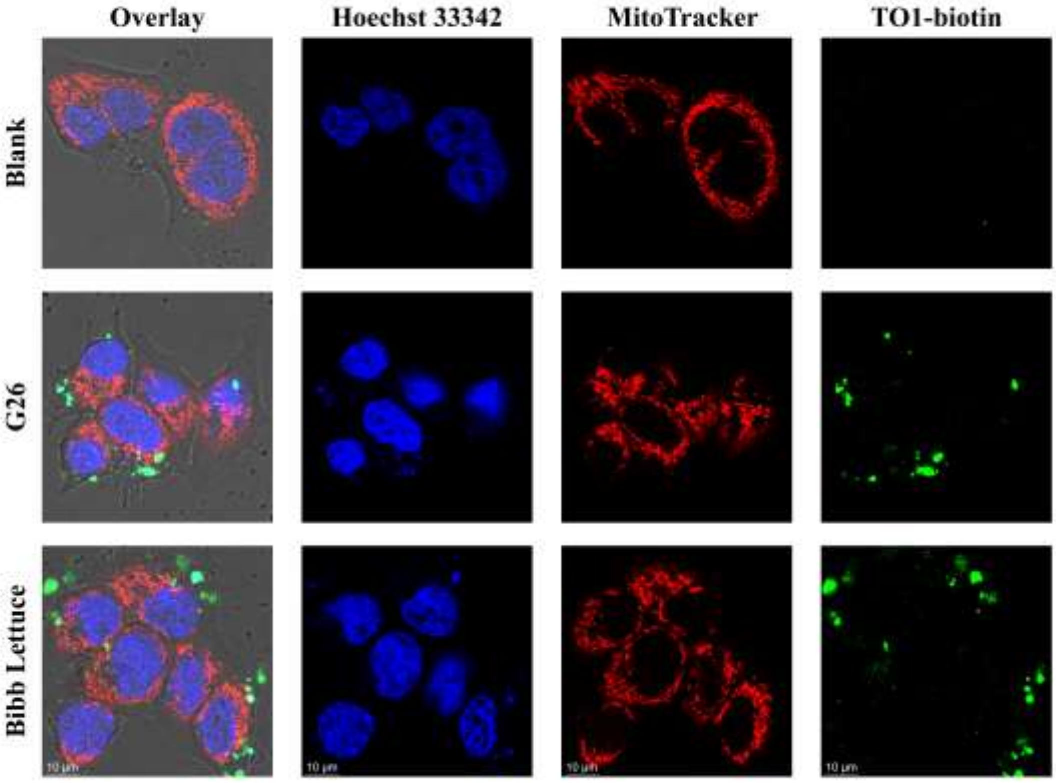

Fig. S7 Co-localisation analysis of TO1-biotin with mitochondria.

Representative confocal images of live cells stained with Hoechst 33342 (10 μg/mL), TO1-biotin (1 μM) and MitoTracker Red CMXRos (100 nM). Blank: no aptamer. Bibb Lettuce: 1 μM Bibb Lettuce aptamer. G26**:** 1 μM **G26** aptamer.

The corresponding quantitative co-localisation data were presented in **Table S2**.

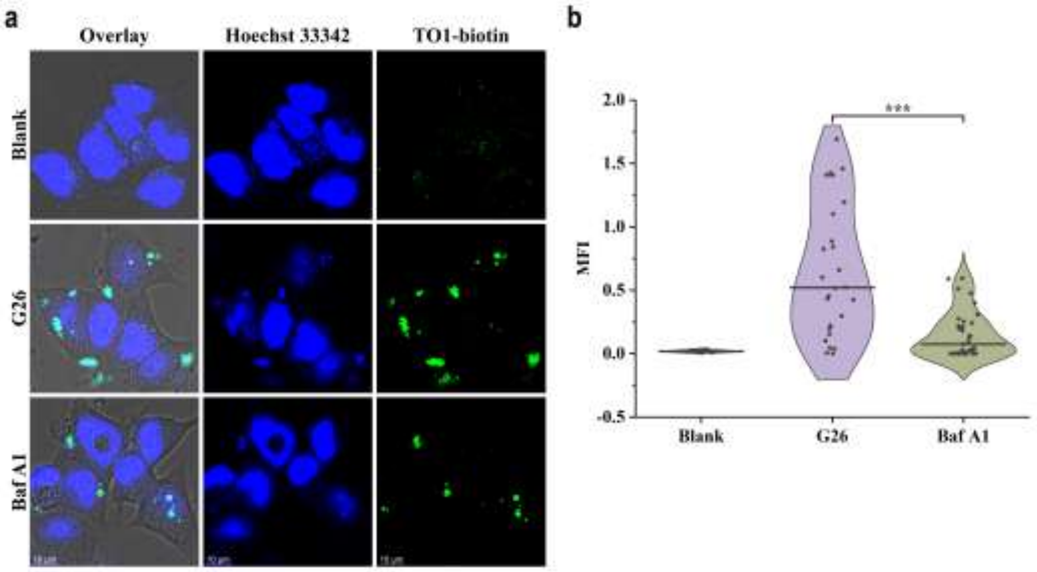

Fig. S8 Endosomal escape inhibition experiment using the **G26** probe.

(a) Representative CLSM images of MCF-7 cells treated with the **G26** aptamer. Blank: Negative control group, without aptamer transfection; **G26**: Positive control group, transfected with 1.0 μM **G26** probe. Baf A1: pre-incubated with culture medium containing 100 nM Bafilomycin A1 for 1 h, then transfected with 1.0 μM G26 probe under Bafilomycin A1 condition. After transfection, all groups were stained with 1 μM TO1-biotin and 10 μg/mL Hoechst 33342 before imaging. (b) Quantitative analysis of single-cell fluorescence intensity across the groups. Data represented as median (black line) with individual data points. Statistical significance was determined by Mann–Whitney *U* test: ***, $p < 0.001$.

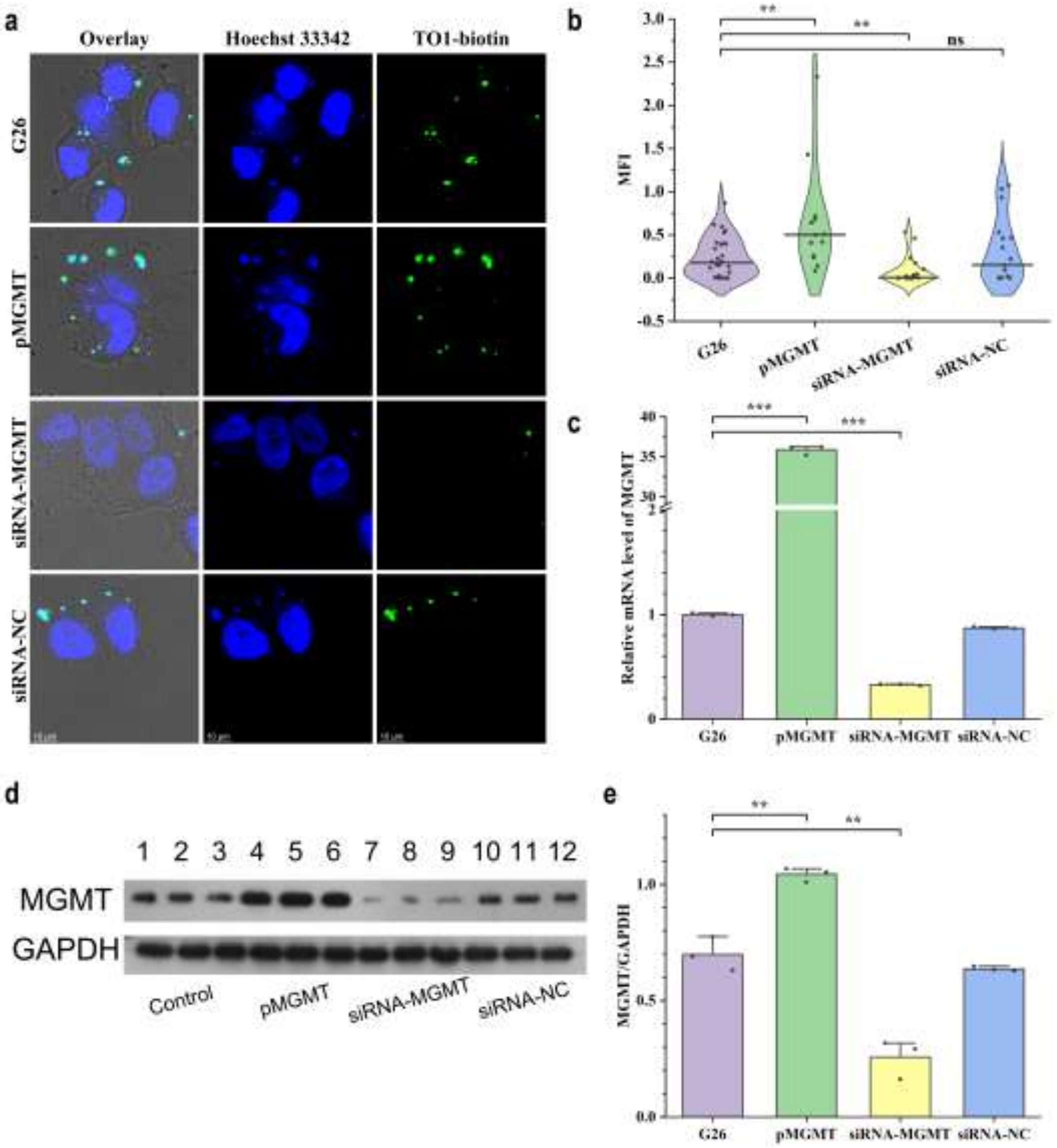

Fig. S9 MGMT overexpression and knockdown validation using the **G26** probe.

(a) Representative CLSM images of MCF-7 cells treated with the **G26** aptamer. **G26**: Control group, transfected with 1.0 μM **G26** probe without plasmids/siRNA pre-transfection. pMGMT: pre-transfected 2 μg/mL MGMT plasmid, then transfected with 1.0 μM **G26** probe. siRNA-NC: pre-transfected 50 nM siRNA (negative control), then transfected with 1.0 μM **G26** probe. siRNA-MGMT: pre-transfected 50 nM siRNA (MGMT), then transfected with 1.0 μM **G26** probe. After transfection, all groups were stained with 1 μM TO1-biotin and 10 μg/mL Hoechst 33342 before imaging. (b) Quantitative analysis of single-cell fluorescence intensity across the groups. Data represented as median (black line) with individual data points. Statistical significance was determined by Mann–Whitney *U* test: **, $p < 0.01$; ns, not significant ($p > 0.05$).

(c) Relative mRNA level of MGMT across the groups. (d) Representative Western blot analysis of MGMT across the groups, with the no plasmids/siRNA pre-transfection group as the normal control group. (e) Quantitative analysis of band intensities following densitometric quantification, normalised to GAPDH. Statistical significance was determined by ANOVA Oneway test: ***, $p < 0.001$.

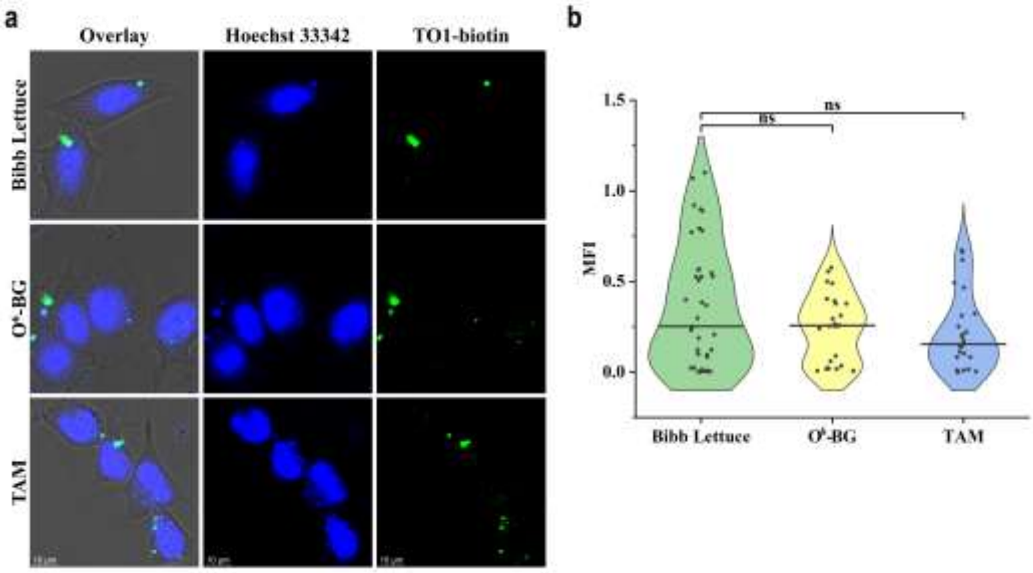

Fig. S10 Inhibitor specificity control in living cells using the Bibb Lettuce aptamer. (a) Representative CLSM images of MCF-7 cells treated with the Bibb Lettuce aptamer in the presence of MGMT inhibitors. Bibb Lettuce: Control group, transfected with 1.0 μM Bibb Lettuce aptamer. O^6-BG: pre-incubated with culture medium containing 1 μM O^6-BG for 40 h, then transfected with 1.0 μM Bibb Lettuce aptamer; TAM: pre-incubated with culture medium containing 25 μM tamoxifen for 40 h, then transfected with 1.0 μM Bibb Lettuce aptamer. After transfection, all groups were stained with 1 μM TO1-biotin and 10 μg/mL Hoechst 33342 before imaging. (b) Quantitative analysis of single-cell fluorescence intensity across the groups. Data represented as median (black line) with individual data points. Statistical significance was determined by Mann–Whitney U test: ns, not significant ($p > 0.05$).

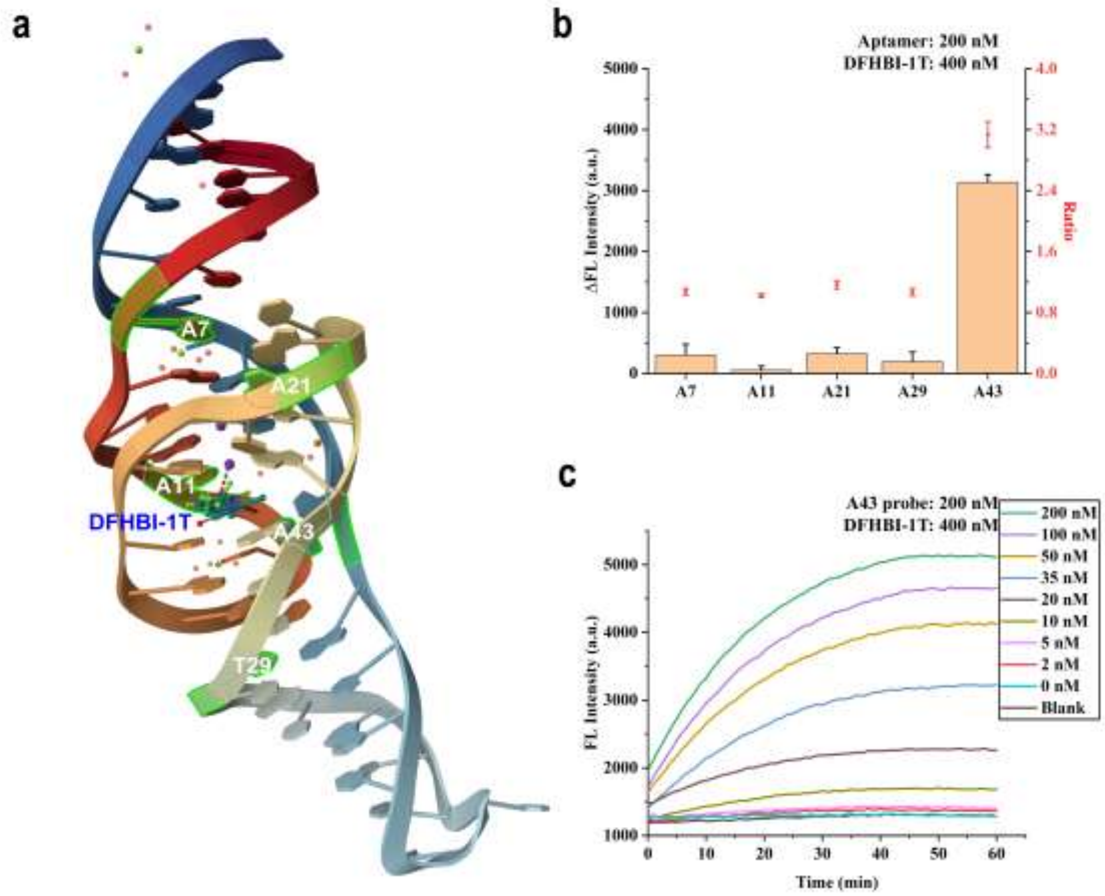

Fig. S11 Recognition of AlkBH2 by DFHBI-1T@aptamer complexes labelled with 1-meA.

(a) The relative positions of selected 5 adenines and dye molecule (DFHBI-1T) in the Bibb Lettuce aptamer. (b) Fluorescence increases and proportion values of the AlkBH2 (100 nM) adding group relative to the non-adding group. (c) Fluorescence values of **A43** probe at different concentrations of AlkBH2 over the course of 1 hour.

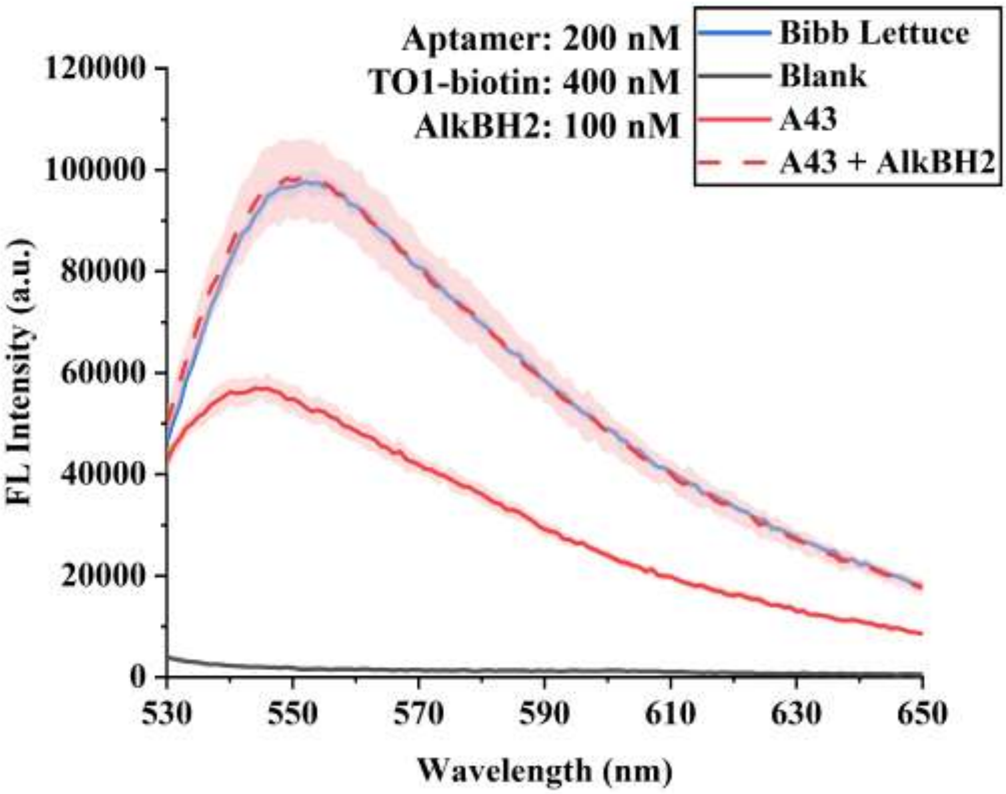

Fig. S12 Fluorescence emission spectra of the **A43** aptamer incubated with TO1-biotin for 1 hour with or without the addition of AlkBH2 (100 nM).

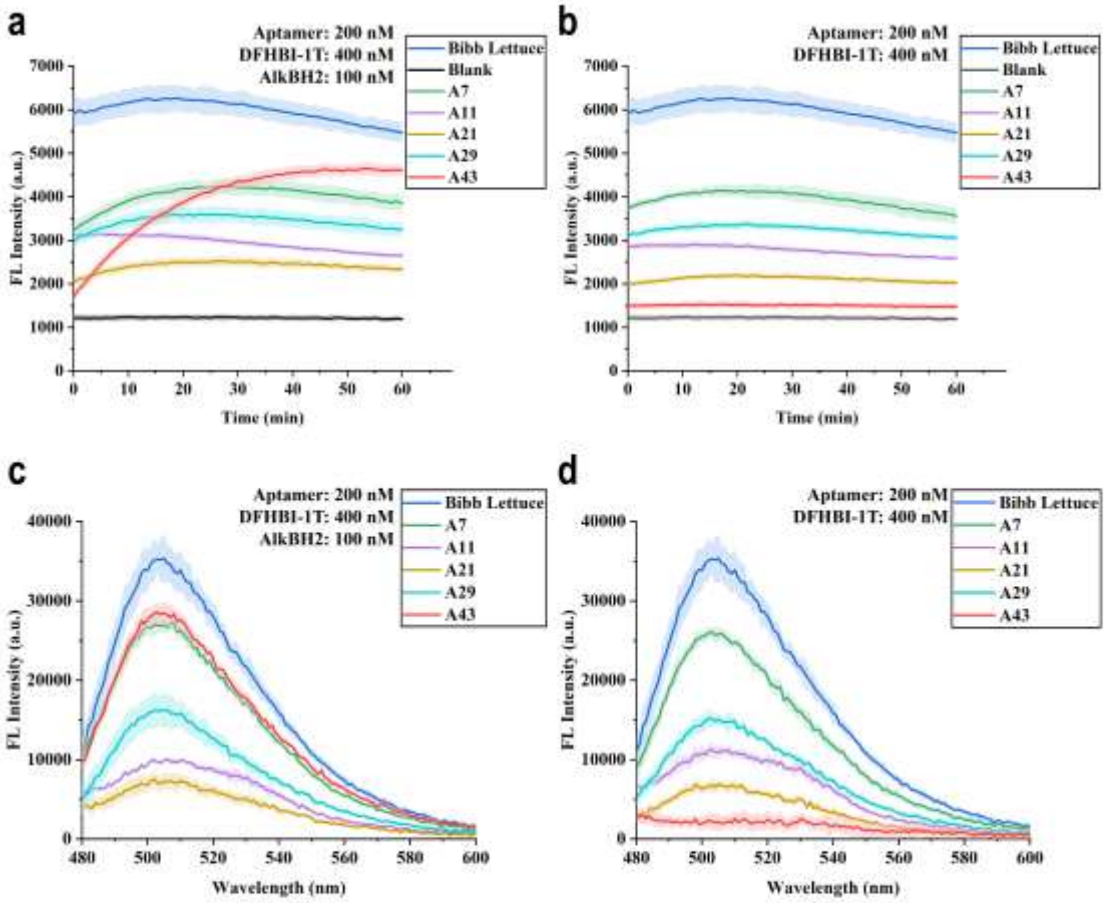

Fig. S13 Fluorescence values and fluorescence emission spectra of 1-meA-labelled aptamer with and without the addition of AlkBH2 within 1 hour.

(a) Fluorescence values of 1-meA-labelled aptamer with the addition of 100 nM AlkBH2 within 1 hour; (b) Fluorescence values of 1-meA-labelled aptamer without the addition of AlkBH2 within 1 hour; (c) Fluorescence emission spectra of 1-meA-labelled aptamer with the addition of 100 nM AlkBH2 after 1 hour; (d) Fluorescence emission spectra of 1-meA-labelled aptamer without the addition of AlkBH2 after 1 hour. The fluorescence emission spectra for each group are presented relative to the Blank group as baseline. The error bars show the standard deviation (n = 3).

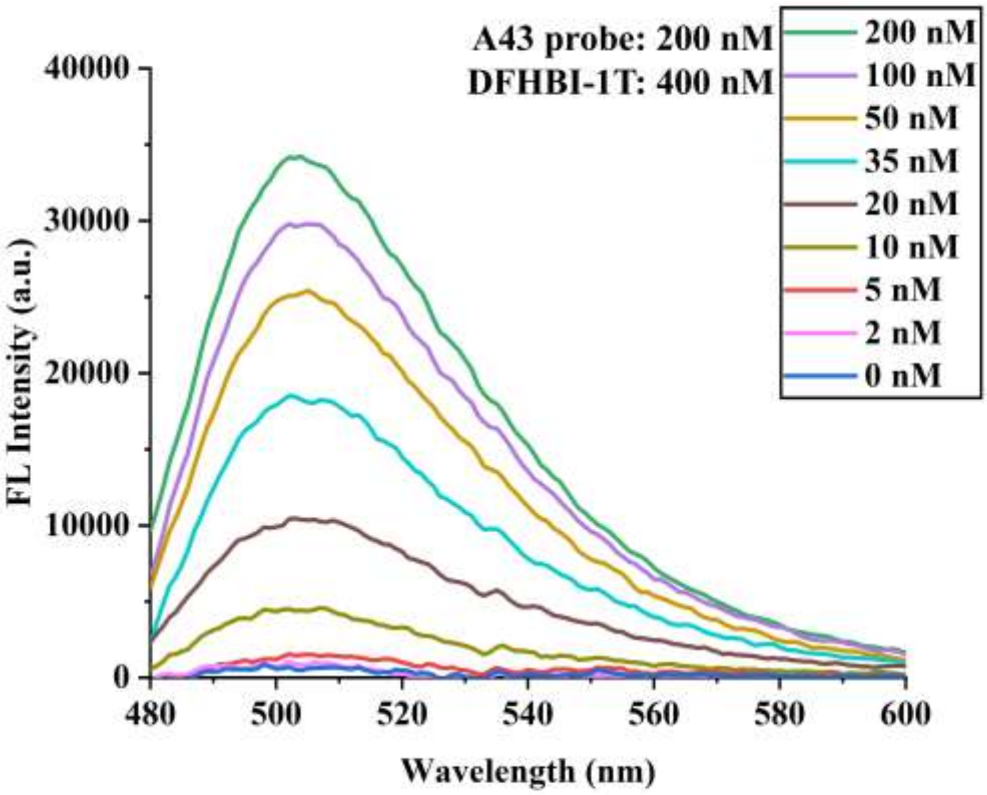

Fig. S14 Fluorescence emission spectra of the **A43** probe with the addition of different concentrations of AlkBH2 after 1 hour.

The fluorescence results for each group are presented relative to the Blank group as baseline. The spectrum has been smoothed.

Reference

39. W. L. Jorgensen, J. Chandrasekhar, J. D. Madura, R. W. Impey and M. L. Klein, *J. Chem. Phys.*, 1983, **79**, 926–935.

40. J. Wang, R. M. Wolf, J. W. Caldwell, P. A. Kollman and D. A. Case, *J. Comput. Chem.*, 2004, **25**, 1157–1174.

41. M. Zgarbova, J. Šponer, M. Otyepka, T. E. Cheatham, R. Galindo-Murillo and P. Jurečka, *J. Chem. Theory Comput.*, 2015, **11**, 5723–5736.

42. W. F. Van Gunsteren and H. J. C. Berendsen, *Mol. Simulat.*, 1988, **1**, 173–185.

43. C. H. Bennett, *J. Comput. Phys.*, 1976, **22**, 245–268.

44. M. Parrinello and A. Rahman, *J. Appl. Phys.*, 1981, **52**, 7182–7190.

45. J.-P. Ryckaert, G. Ciccotti and H. J. C. Berendsen, *J. Comput. Phys.*, 1977, **23**, 327–341.

46. U. Essmann, L. Perera, M. L. Berkowitz, T. Darden, H. Lee and L. G. Pedersen, *J. Chem. Phys.*, 1995, **103**, 8577–8593.
